# Structures of *Rhodopseudomonas palustris* RC-LH1 complexes with open or closed quinone channels

**DOI:** 10.1126/sciadv.abe2631

**Published:** 2021-01-13

**Authors:** David J. K. Swainsbury, Pu Qian, Philip J. Jackson, Kaitlyn M. Faries, Dariusz M. Niedzwiedzki, Elizabeth C. Martin, David A. Farmer, Lorna A. Malone, Rebecca F. Thompson, Neil A. Ranson, Daniel P. Canniffe, Mark J. Dickman, Dewey Holten, Christine Kirmaier, Andrew Hitchcock, C. Neil Hunter

**Affiliations:** 1 Department of Molecular Biology and Biotechnology, University of Sheffield, Sheffield, S10 2TN, UK.; 2 Materials and Structural Analysis, Thermo Fisher Scientific, Achtseweg Noord 5, 5651 GG Eindhoven, Netherlands.; 3 Department of Chemical and Biological Engineering, University of Sheffield, Sheffield, S1 3JD, UK.; 4 Department of Chemistry, Washington University in St. Louis, St. Louis, MO 63130, USA.; 5 Center for Solar Energy and Energy Storage, Washington University in St. Louis, St. Louis, MO 63130, USA.; 6 Department of Energy, Environmental and Chemical Engineering, Washington University in St. Louis, St. Louis, MO 63130, USA.; 7 Astbury Centre for Structural Molecular Biology, School of Molecular and Cellular Biology, University of Leeds, Leeds, LS2 9JT, UK.; 8 Department of Biochemistry and Systems Biology, Institute of Systems, Molecular and Integrative Biology, University of Liverpool, Liverpool, L69 7ZB, UK.

## Abstract

The reaction-center light-harvesting complex 1 (RC-LH1) is the core photosynthetic component in purple phototrophic bacteria. We present two cryo–electron microscopy structures of RC-LH1 complexes from *Rhodopseudomonas palustris*. A 2.65-Å resolution structure of the RC-LH1_14_-W complex consists of an open 14-subunit LH1 ring surrounding the RC interrupted by protein-W, whereas the complex without protein-W at 2.80-Å resolution comprises an RC completely encircled by a closed, 16-subunit LH1 ring. Comparison of these structures provides insights into quinone dynamics within RC-LH1 complexes, including a previously unidentified conformational change upon quinone binding at the RC Q_B_ site, and the locations of accessory quinone binding sites that aid their delivery to the RC. The structurally unique protein-W prevents LH1 ring closure, creating a channel for accelerated quinone/quinol exchange.

## Teaser

High-resolution structures of reaction-center light-harvesting 1 complexes provide new insights into quinone dynamics.

## INTRODUCTION

Photosynthesis provides the energy that sustains almost all life on Earth and has enormous potential for solar-powered biotechnology. As well as contributing to global photosynthesis, purple phototrophic bacteria display a wide diversity of energy modes and metabolic capabilities; they can dispense with photosynthesis and grow as heterotrophs in the dark, they can fix nitrogen and CO_2_, produce hydrogen, and degrade aromatic compounds (*1*–*3*). To provide energy for these processes, light must be rapidly and efficiently converted into chemical energy, a process that starts when light-harvesting antenna complexes absorb light and transfer the captured energy to the reaction center (RC), initiating a charge separation (*4*–*7*). The basic unit of photosynthesis in purple phototrophic bacteria consists of a type 2 RC surrounded by light-harvesting complex 1 (LH1), forming the RC-LH1 core complex. LH1 is formed from curved arrays of αβ heterodimers, each of which binds two bacteriochlorophyll (BChl) *a* molecules and one or two carotenoids (*8*–*12*). The simplest LH1 antenna consist of 16 or 17 αβ heterodimers encircling the RC in a closed ring (*9*–*13*), but in other core complexes transmembrane polypeptides interrupt the continuity of the encircling LH1 to facilitate quinol/quinone diffusion between the RC and the cytochrome *bc*_1_ complex (*11*, *13*–*15*). The purple phototroph *Rhodopseudomonas* (*Rps*.) *palustris* is a model organism for understanding the energy and electron transfers that underpin photosynthesis. The first crystallographic structure of the *Rps. palustris* RC-LH1 complex was modeled as a RC surrounded by a 15-heterodimer LH1 ring interrupted by an unknown protein assigned as “protein-W” (*14*). Protein-W was later identified as RPA4402, an uncharacterized 10.5-kDa protein with three predicted transmembrane helices (TMHs) (*16*). We propose to rename the rpa4402 gene encoding protein-W as *pufW*, in keeping with the nomenclature used for the genes encoding RC-L, M (*pufL*, *pufM*), and LH1 α, β (*pufA*, *pufB*) subunits. Intriguingly, protein-W was only present in approximately 10% of RC-LH1s, revealing that *Rps. palustris* produces two distinct RC-LH1 complexes. Here, we report the high-resolution cryo–electron microscopy (cryo-EM) structures of both core complexes, one with protein-W and 14 αβ heterodimers and the other with no protein-W and a closed 16-heterodimer LH1 ring. Our structures represent a step change in the understanding of the RC-LH1 complexes of *Rps. palustris* because we have analyzed homogeneous populations of each variant with sufficient resolution for the unambiguous assignment of each polypeptide, as well as bound pigments and associated lipids and quinones. Comparison of these structures suggests that the three TMH protein-W, not found in any other RC-LH1 complex analyzed to date, creates a quinone channel for accelerated quinone/quinol exchange. Multiple conserved lipid and quinone binding sites are identified, and we reveal a novel conformational change upon quinone binding to the RC, which may be applicable to the photosystem II (PSII) RCs of oxygenic phototrophs. Our findings provide new insights into the dynamics of quinone/quinol binding and exchange in the RC-LH1 core complexes of purple phototrophic bacteria.

## RESULTS AND DISCUSSION

### Overall structure of the two core complexes

To facilitate a detailed investigation of the two complexes found in *Rps. palustris*, we biochemically isolated each RC-LH1. Complexes lacking protein-W were purified from a strain where the *pufW* gene was deleted (hereafter Δ*pufW)* (*16*) allowing only one type of RC-LH1 complex to be produced. Complexes containing protein-W were produced from a strain where protein-W was modified with a 10× His tag at its C terminus, permitting efficient separation of the protein-W containing complexes from the majority lacking protein-W (*16*) by immobilized metal affinity chromatography (IMAC).

**Fig. 1:**
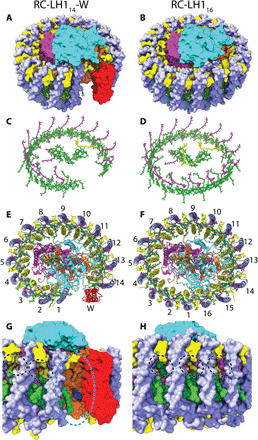
Overall architecture of RC-LH1_14_-W and RC-LH1_16_ complexes. (**A** and **B**) The complexes in surface representation. (**C** and **D**) Bound pigments in stick representation. (**E** and **F**) The complexes viewed from the cytoplasmic face with peptides in cartoon representation and LH1 subunits numbered clockwise from the protein-W gap [consistent with numbering for the *Rba. sphaeroides* complex (*13*)]. Protein subunits are colored in yellow for LH1-α, light blue for LH1-β, red for protein-W, cyan for RC-H, orange for RC-L, and magenta for RC-M. Cofactors are shown in stick representation with BChl and BPh *a* molecules in green, carotenoids in purple, and UQ_10_ molecules in yellow. (**G** and **H**) Zoomed views of the protein-W gap in the RC-LH1_14_-W complex (G) and equivalent region of the RC-LH1_16_ complex (H). Cofactors are shown in space-filling representation with sequestered quinones displayed in blue. The protein-W gap is highlighted with a blue dashed line in (G), and small pores for quinone/quinol diffusion across the LH1_16_ ring are highlighted with black dashed lines in (H).

As shown in Fig. 1, both complexes contain a three-subunit RC (RC-L, RC-M, and RC-H) surrounded by an LH1 antenna. The 2.80-Å structure of the complex that lacks protein-W shows 16 αβ heterodimers forming a closed LH1 ring completely encircling the RC, hereafter referred to as the RC-LH1_16_ complex. The 2.65-Å structure of the complex containing protein-W has a 14-heterodimer LH1 interrupted by protein-W, hereafter RC-LH1_14_-W.

### LH1 structure

Figure 1 (A and B) shows an RC enclosed by open or closed arrays of LH1 αβ heterodimers, each of which binds two BChls and one carotenoid (Fig. 1, C and D). Previous studies have shown that the LH1 complexes of *Rps.* species contain mixed populations of carotenoids in the spirilloxanthin biosynthesis pathway (*17*). However, spirilloxanthin is the dominant carotenoid and gave a satisfactory fit to the densities, so we chose to model spirilloxanthin in all LH1 binding sites. The α- and β-polypeptides are single TMHs with short membrane–extrinsic regions (Fig. 1, A, B, E, and F). The α-polypeptides were resolved from Met^1^ to Ala^46^ in both complexes, although no density was observed for the C-terminal 17 residues. The β-polypeptides were resolved from Gly^4^ to Tyr^52^ in RC-LH1_16_ and Ser^5^ to Tyr^52^ in RC-LH1_14_-W with no observed density for the 3 or 4 N-terminal or 13 C-terminal residues (fig. S1). Mass spectrometry of mixed RC-LH1 complexes prepared from the wild-type strain showed that the missing regions are the result of heterogeneous cleavage of these peptides (figs. S1 and S2). N-terminal formylation (f) of α-Met1 was also observed. This analysis shows that the α-polypeptides consist of residues fMet^1^ to Asp^42^/Ala^46^/Ala^47^/Ala^50^, and β-polypeptides consist of residues Ser^2^ to Ala^53^, in excellent agreement with the cryo-EM density maps.

**Fig. 2:**
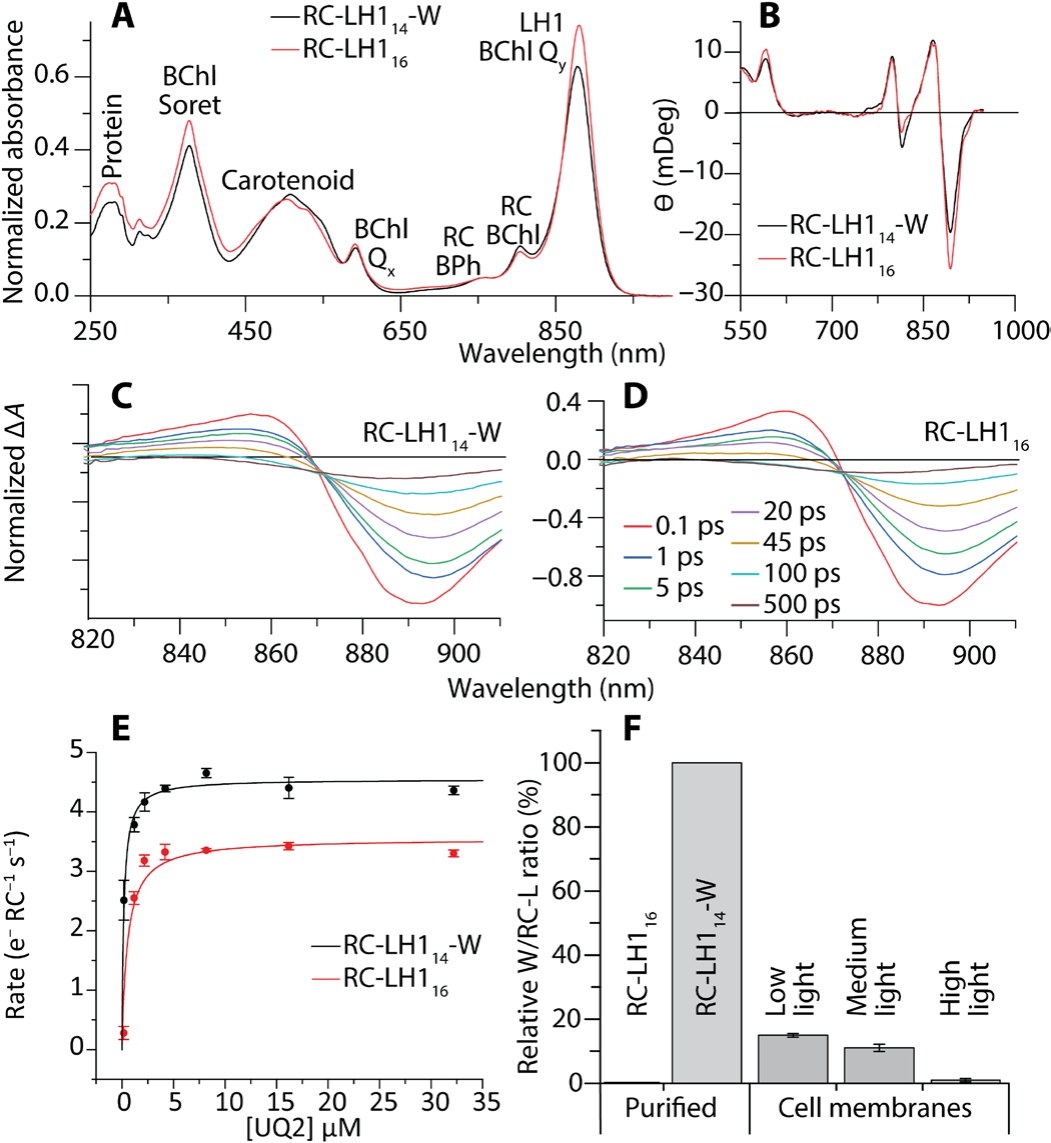
Spectral and biochemical analyses of RC-LH1_14_-W and RC-LH1_16_ complexes. (**A**) Ultraviolet/Vis/NIR absorption spectra with peaks labeled with their corresponding pigments and normalized to the BPh peak at 775 nm. (**B**) Circular dichroism spectra normalized to BChl absorbance at 805 nm. (**C** and **D**) Selected Δ*A* spectra from time-resolved absorption spectroscopy of the RC-LH1_14_-W complex (C) and RC-LH1_16_ complex (D). For better comparability, all spectra are normalized to a ∆*A* of −1 at 0.2 ps. (**E**) The rate of cytochrome *c*_2_ oxidation upon illumination in the presence of various concentrations of UQ_2_ (see fig. S8 for raw data). (**F**) Ratios of protein-W and the RC-L subunit in purified complexes and isolated membranes from cells grown under low, medium or high intensity illumination (10, 30, or 300 μM m^−2^ s^−1^, respectively). Protein levels were determined by SDS–polyacrylamide gel electrophoresis and immunodetection (see fig. S9 for raw data). Ratios were determined relative to the purified RC-LH1_14_-W complex, which has a 1:1 RC-L to protein-W stoichiometry.

Coordination by α-His^29^ and β-His^36^ positions the BChls in a face-to-face arrangement; each αβ heterodimer assembles with its neighbors to form an array of excitonically coupled pigments in an open (RC-LH1_14_-W) or closed (RC-LH1_16_) ring surrounding the RC (Fig. 1, C and D). The 880-nm absorption of RC-LH1_16_ was redshifted by 3 nm relative to the 877 nm band of RC-LH1_14_-W (Fig. 2A). However, the circular dichroism spectra were almost identical (Fig. 2B) suggesting that, despite apparent differences between open and closed rings, the local environments of the BChls are very similar. The absorption redshift may be a result of reduced thermal motion and increased stability upon ring closure (*18*, *19*), alterations to pigment coupling caused by ring closure (*20*, *21*), or a combination of these two effects (*11*).

BChls at position 1 within the distorted αβ_14_ ring of RC-LH1_14_-W (Fig. 1, A, C, and E) are 6.8 Å closer to the RC primary donor (P) than the equivalent BChls in RC-LH1_16_ (Fig. 1, B, D, and F, and fig. S3); however, transient absorption dynamics of the two complexes reveal that the time constants for excitation energy transfer from LH1 to the RC were similar at 40 ± 4 and 44 ± 3 ps for RC-LH1_14_-W and RC-LH1_16_, respectively (Fig. 2, C and D, fig. S4, and table S2). There were also no notable differences in electron transfer within the RCs (fig. S5 and associated Supplementary Text). We suspect that the close correspondence in energy transfer times between LH1 and RC-P arises because the majority of the BChls in both LH1 rings have similar distances, angles, and site energies. It appears that exploring the LH1 energy landscape to reach the distance minimum is not faster than direct energy transfer from a suboptimal site to the RC. It is also possible that the open LH1 ring in RC-LH1_14_-W is subject to thermal motions not apparent in the cryo-state used for structural analysis and that, at room temperature, conformations of the αβ_14_ ring exist with longer interpigment distances from BChls at position 1 to the RC.

**Fig. 3:**
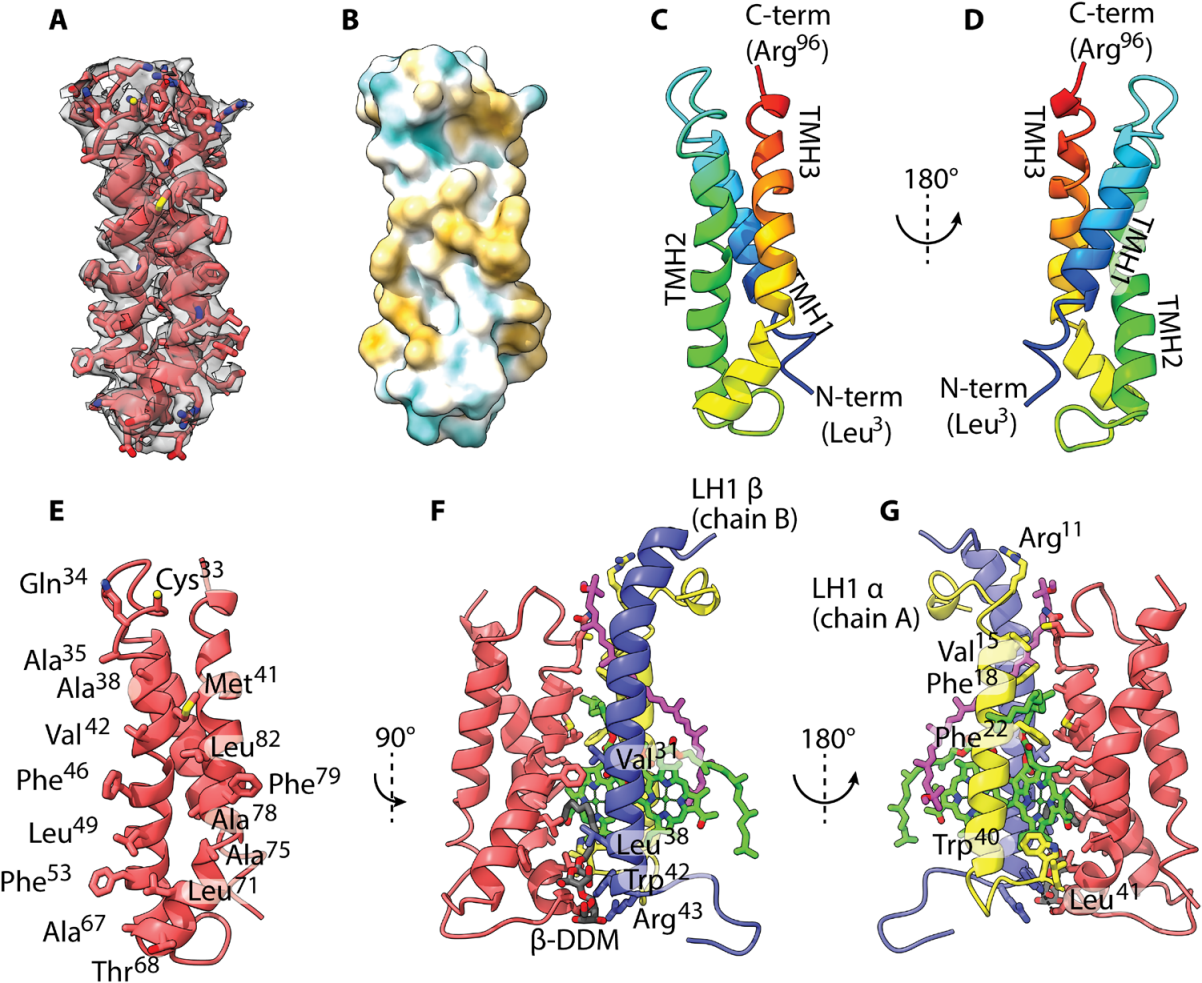
Structure of protein-W and interactions with LH1. (**A**) Protein-W viewed facing the interface with LH1 αβ14 in cartoon representation with side chains as sticks (red) shown within its portion of the electrostatic potential map (transparent gray surface at a contour level of 0.13). (**B**) Protein-W in surface representation colored by hydrophobicity. Polar and charged regions are shown in cyan, hydrophobic regions are shown in white, and strongly hydrophobic regions are in orange. (**C** and **D**) Protein-W in cartoon representation in the same orientation as in (A) (C) and rotated 180° (D). Resolved residues are in a rainbow color scheme according to position in the sequence with the N terminus in blue and C terminus in red. (**E**) Protein-W in the same view as in (A) with residues at the protein-W:LH1 interface in stick representation with accompanying labels. (**F**) Protein-W rotated 90° relative to (E) with LH1 αβ14 in cartoon representation and interface residues in stick representation. Highlighted residues from the β polypeptide are labeled. Cofactors are shown as sticks with coloring matching Fig. 1, and the resolved β-DDM is shown in gray with oxygens in red. (**G**) The view in (F) rotated 180° with highlighted residues from the α polypeptide labeled.

The RC-LH1_16_ complex contains 32 BChls and 16 carotenoids in an overall arrangement similar to the closed-ring RC-LH1 complexes from *Thermochromatium* (*Tch.*) *tepidum* [Protein Data Bank (PDB) ID 5Y5S] (*9*), *Thiorhodovibrio* (*Trv.*) strain 970 (PDB ID 7C9R) (*12*), and *Blastochloris* (*Blc.*) *viridis* (PDB ID 6ET5) (*10*). When aligned, only small deviations in the positions of the αβ heterodimers, particularly 1 to 5, 15, and 16, were observed (fig. S6). The presence of protein-W has a marked effect on the LH1 structure; its three TMHs are connected by short loops, with the N terminus on the lumenal side of the complex and the C terminus on the cytoplasmic side (Figs. 1A and 3, A to D). Protein-W is largely hydrophobic (Fig. 3B) with the transmembrane surface formed by TMH2 and TMH3 interacting with LH1 αβ-14 (Fig. 3, B and E to G). The interface is composed mainly of Phe, Leu, and Val residues within the transmembrane region, which pack against hydrophobic amino acids and pigments of αβ-14. Some polar residues also contribute to the interaction, including a hydrogen bond between W-Thr^68^ and β-Trp^42^ on the lumenal face of the complex (Fig. 3, F and G). On the cytoplasmic face, Gln^34^ is in close proximity to the keto-group of the αβ-14 carotenoid. In addition, a molecule of *n*-dodecyl β-d-maltoside (β-DDM) detergent was resolved with its hydrophobic tail extending into the interface between protein-W and αβ-14, where a lipid tail may be located in vivo. We also note that the resolved C-terminal regions of protein-W and RC-H are in close proximity but not within range for specific interactions to form (Fig. 1, A and E). However, it is possible that there are interactions in the unresolved C-terminal amino acids of these two proteins that may provide the mechanism for recruitment of protein-W during assembly of the RC-LH1_14_-W complex.

**Fig. 4:**
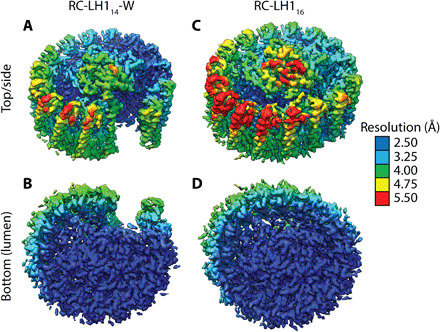
Density maps colored by local resolution as determined with the Relion local resolution tool. The maps for RC-LH1_14_-W (**A** and **B**) and RC-LH1_16_ (**C** and **D**) are shown from the same top/side view in Fig. 1 (A and B) (A and C) and from the lumenal surface of the complex (B and D). The color key is shown on the right.

Protein-W replaces one αβ heterodimer, the 15th in Fig. 1F, preventing closure of the ring and tilting the first three αβ heterodimers. The largest tilt of 25° to 29° relative to the membrane normal is observed for the first αβ-1 heterodimer (Fig. 1, A and E), in stark contrast to the 2° to 8° tilt of αβ-1 in RC-LH1_16_ (Fig. 1, B and F). The second and third heterodimers are tilted by 12° to 22° and 5° to 10°, respectively. Tilting of αβ-1 excludes a second αβ pair (which would have corresponded to the 16th αβ in Fig. 1F) due to steric hindrance by the RC, creating a distinct gap in the LH1 ring (Fig. 1, A and E). Along with the loss of four BChls and two carotenoids due to the absence of two αβ heterodimers, no carotenoid binds to the distorted αβ-1 subunit, resulting in an LH1_14_-W ring containing 13 carotenoids and 28 BChls. Local resolution estimates for the two complexes in the region of αβs 1 to 7 were lower than for the rest of the LH1 ring, which could reflect inherent plasticity of LH1 subunits adjacent to the RC Q_B_ site (Fig. 4).

The only other characterized core complex with a 1:14 RC:LH1 stoichiometry is the *Rhodobacter* (*Rba.*) *sphaeroides* RC-LH1-PufX dimer (*13*). However, protein-W and PufX share no significant homology and have distinct effects on their respective LH1 structures. PufX is a single TMH with an N-terminal cytoplasmic domain that interacts with the cytoplasmic side of the RC-H subunit (*13*) in a location corresponding to *Rps. palustris* LH1_16_ αβ-16. PufX creates a channel for quinone/quinol exchange between RC-LH1 and the cytochrome *bc*_1_ complex and is present in all *Rba. sphaeroides* core complexes (*13*). Although the monomer-monomer interface in the *Rba. sphaeroides* RC-LH1-PufX dimer is in the position that protein-W binds in the RC-LH1_14_-W, the gaps induced by PufX and protein-W are in equivalent positions (fig. S7A). The gap in RC-LH1_14_-W also aligns with the hypothesized quinone channel of *Roseiflexus castenholzii* LH1 *(**8**)*, which is formed by peptides bearing no relation to protein-W or PufX (fig. S7B). Further, the quinone channel in the *Blc. viridis* LH1, formed by exclusion of one γ subunit *(**7**)*, is found at a similar position (fig. S7C). Despite being mediated by different proteins, the emergence of these quinone/quinol channels in common locations within RC-LH1 complexes appears to be an example of convergent evolution and suggests that the gap produced by protein-W likely serves as a quinone channel.

The gap in the LH1_14_-W ring allows formation of a continuous membrane region between the internal space of the RC-LH1_14_-W complex and the bulk membrane (Fig. 1G), rather than connection of these two domains by protein pores as in the RC-LH1_16_ complex, similar to those of the closed *Tch*. *tepidum* complex (*22*) (Fig. 1H). As quinone diffusion through the membrane is expected to be faster than diffusion through narrow protein channels, the open LH1_14_-W ring may permit faster RC turnover than the closed LH1_16_ ring, in which quinone access to the RC may be more diffusion limited. To test whether protein-W influences the turnover of quinone by the RC, we performed cytochrome oxidation assays over a range of concentrations of ubiquinone 2 (UQ_2_), an analog of the native UQ_10_ with a shorter isoprene tail (Fig. 2E). Although the presence of sequestered quinone precludes the accurate determination of an apparent Michaelis constant (fit at 0.2 ± 0.1 μM and 0.5 ± 0.2 μM for RC-LH1_14_-W and RC-LH1_16_, respectively), the maximum rate of RC-LH1_14_-W (4.6 ± 0.2 e^−^ RC^−1^ s^−1^) was 28 ± 5% greater than that of RC-LH1_16_ (3.6 ± 0.1 e^−^ RC^−1^ s^−1^).

We originally estimated that protein-W is present in ~10% of core complexes (*16*); here, occupancies of 15 ± 0.6%, 11 ± 1%, and 0.9 ± 0.5% were found for low-, medium-, and high-light grown cells, respectively (Fig. 2F). Comparative quantification by mass spectrometry shows that relative abundance of protein-W is not lowered by addition of a histidine-tag relative to the wild-type strain (*P* = 0.59), so these levels are not an artefact of modifying protein-W (fig. S10). Such low occupancies of protein-W in the RC-LH1 complexes could nevertheless allow some RCs to turn over at an accelerated rate, alleviating slower quinone/quinol exchange in RC-LH1_16_ complexes. We note that the high-light occupancy is at odds with recent transcriptomics data suggesting increased expression of the *pufW* gene under strong illumination (fig. S11) (*23*). The discrepancy between *pufW* transcription and incorporation of protein-W into RC-LH1 complexes is perplexing and may reflect complex regulation of this protein.

### Structurally defined lipids and UQs

**Fig. 5:**
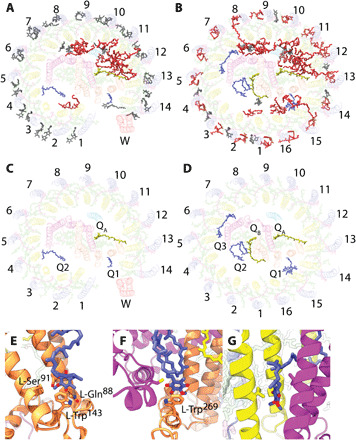
Structurally defined lipids and quinones. (**A** and **B**) RC-LH1_14_-W (A) and RC-LH1_16_ (B) polypeptides are shown in cartoon representation and pigments as sticks, using the color scheme in Fig. 1. Lipids are shown in red, and detergents are shown in gray. UQs bound to the RC Q_A_ and Q_B_ sites are in yellow, and sequestered UQs are in blue. (**C** and **D**) The same view as in (A) and (B) with the lipids omitted. (**E** to **G**) Zoomed views of Q1 (E), Q2 (F), and Q3 (G) from RC-LH1_16_ with interacting side chains in stick representation. Hydrogen bonds are shown with a black dashed line.

In RC-LH1_14_-W, 6 cardiolipin (CDL), 7 phosphatidylcholine (POPC), 1 phosphatidylglycerol (POPG), and 29 β-DDM molecules were assigned, and 6 CDL, 24 POPC, 2 POPG, and 12 βDDM were modeled in RC-LH1_16_ (Fig. 5, A and B). In both structures, the CDLs were almost exclusively located on the cytoplasmic side of the complex, while POPC, POPG, and β-DDM were mostly on the lumenal side. Two lipid and detergent molecules are sequestered within the αβ-1 to αβ-6 region of the RC-LH1_14_-W complex (Fig. 5A), with five in the equivalent area of RC-LH1_16_ (Fig. 5B). Many more lipids, mostly CDL, are found on the opposite side of the complex, packed between the RC and αβ-7 to αβ-13 (Fig. 5, A and B). Additional structurally resolved lipids and detergents are located on the outside of the LH1 ring with the well-resolved acyl chains extending between LH1 subunits, tentatively assigned as β-DDM in RC-LH1_14_-W and a mixture of β-DDM and POPC in RC-LH1_16_. The similarity in the positions of sequestered lipids and detergents in our structures suggests that these are physiologically relevant binding sites (fig. S12A). There is also good agreement with the positions of equivalent molecules in the *Tch. tepidum* and *Trv*. strain 970 RC-LH1s (fig. S12, B to E) (*9*, *12*), and hydrogen-bonding residues to the lipid head groups appear reasonably well conserved in sequence alignments (fig. S13), indicating that, in addition to a conserved CDL bound to the RC (*24*), these sites may be conserved in RC-LH1 complexes.

In RC-LH1_16_, both the RC Q_A_ and Q_B_ UQs, which participate in electron transfer during charge separation, were resolved within their binding sites. However, in RC-LH1_14_-W, the Q_B_ quinone was not resolved, which will be discussed in detail below. In addition to Q_A_ and Q_B_ quinones, two sequestered UQ molecules (located in the space between the RC and the LH1 ring) were tentatively assigned in the RC-LH1_14_-W structure, based on their well-resolved head groups (Q1 and Q2 in Fig. 5C). Two isoprene units were assigned for Q1, and the density map resolves the full 10-isoprene tail for Q2. In the RC-LH1_16_ structure, three sequestered UQ_10_ molecules were resolved (Q1 to Q3, Fig. 5D), all of which had clear density for the entire tail (Fig. 5, D to G). There is excellent agreement in the positions of the quinone head groups for Q1 and Q2 in both structures (fig. S12F), which interact exclusively with the RC. Q1 is located at the entrance to the W-gap of RC-LH1_14_-W (Figs. 1G and 5, C, D, and E), and Q2 is located close to the Q_B_ binding site (Fig. 5, C, D, and F). The conserved L-Trp^143^ and L-Trp^269^ residues are in close proximity to Q1 and Q2 and provide potential π-stacking interactions (Fig. 5, E and F, and fig. S12). L-Gln^88^, 3.0 Å from the distal oxygen of Q1, provides a strong hydrogen bond (Fig. 5E); this residue is conserved in all but the most distantly related RCs (fig. S13). L-Ser^91^, which is conservatively substituted for Thr in most other RCs (fig. S13), is 3.8 Å from a methyl oxygen of Q1 and may provide a weak hydrogen bond (Fig. 5E). Q3 does not appear to make specific interactions but is located in a hydrophobic region between the RC-M subunit and LH1-α subunits 5 to 6 (Fig. 5, D and G). Sequestered quinones at or near to Q1, Q2, and Q3 have also been resolved in the *Tch. tepidum*, *Trv.* strain 970, and *Blc. viridis* structures (*9*, *10*, *12*), pointing to conserved accessory quinone binding sites within RC-LH1 complexes (fig. S12G). The five resolved UQs in RC-LH1_16_ are in good agreement with the 5.8 ± 0.7 per complex determined by high-performance liquid chromatography (HPLC), while the three in RC-LH1_14_-W is lower than the measured 6.2 ± 0.3 (fig. S14), suggesting the presence of further unresolved UQ molecules in the structure.

### Reaction center

The pseudosymmetric L and M polypeptides each comprise five TMHs and form a heterodimer that binds one BChl dimer, two BChl monomers, two bacteriopheophytin (BPh) monomers, one non-heme iron, and one or two UQ_10_ molecules. A carotenoid is bound in the M-subunit, assigned as *cis*-3,4-dehydrorhodopin by the presence of a hydrogen bond to the terminal keto group and its known accumulation in *Rps.* species (*25*). The membrane-extrinsic domain of RC-H is anchored to the membrane by a single TMH. The overall RC structure is similar to three-subunit RCs of related species, such as *Rba. sphaeroides* (PDB ID: 3I4D). The macrocycles of BChl and BPh, the carotenoid backbone, and the non-heme iron superimpose within the resolution limits of these structures, as do the head groups of UQ_10_ at the Q_A_ site and the Q_B_ quinone of RC-LH1_16_ (fig. S15).

The availability of two RC structures that differ in the occupancy of the Q_B_ site provides a new opportunity to examine the concerted conformational changes that accompany binding of the Q_B_ quinone. In the RC-LH1_16_ complex, the Q_B_ quinone is located in the fully bound “proximal” position (*26*), but RC-LH1_14_-W was resolved without a Q_B_ quinone. The absence of a Q_B_ quinone in RC-LH1_14_-W was unexpected as the complex is active, more so than the RC-LH1_16_ complex that does have a structurally resolved Q_B_ quinone. Although both LH1 rings sequester approximately six quinones, five are resolved structurally in the closed RC-LH1_16_ ring, whereas only three are structurally defined within the open RC-LH1_14_-W ring. This increased structural disorder likely reflects faster turnover at the RC-LH1_14_-W Q_B_ site, more rapid quinone dynamics within the complex, and an increased probability of traversing the LH1 ring. We suggest that the absence of a structurally resolved UQ at the RC Q_B_ site of RC-LH1_14_-W is likely a consequence of a more disordered and more active complex and that the Q_B_ site in RC-LH1_14_-W has been frozen instantaneously in a conformation that reflects this activity, in a particular phase of UQ turnover where the entrance to the Q_B_ site has closed.

**Fig. 6:**
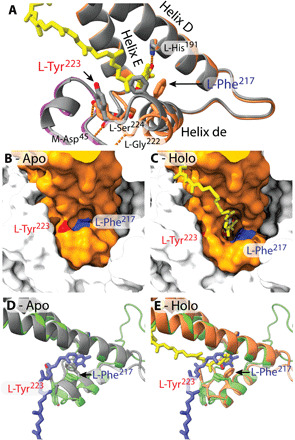
Conformational changes upon quinone binding to the RC Q_B_ site. (**A**) Overlaid cartoons of holo (chain L, orange/chain M, magenta) and apo (gray) structures with key residues displayed in stick representation. UQ_10_ is shown in stick representation in yellow. Dashed lines show hydrogen bonds formed in the holo structure. (**B** and **C**) Surface representations of apo and holo structures, respectively, with L-Phe^217^ highlighted in blue and the side chain oxygen of L-Tyr^223^ in red. Subunit L is orange; and subunits M and H are not colored. (**D** and **E**) The apo (D) and holo (E) RC Q_B_ sites [colored as in (A)], respectively, aligned to the *Thermosynechococcus vulcanus* PSII (green with plastoquinone in blue; PDB ID: 3WU2) (*58*).

The absence of Q_B_ is accompanied by rotation of L-Phe^217^ into a position incompatible with UQ_10_ binding as it would cause a steric clash with the first isoprene unit of the tail (Fig. 6A). Further, major conformational changes are apparent, notably the shifting of helix de (a short helix in the loop between TMHs D and E) on which L-Phe^217^ resides into the Q_B_ binding pocket and a rotation of L-Tyr^223^ (Fig. 6A), breaking the hydrogen bond to the backbone of M-Asp^45^ and closing the entrance of the Q_B_ binding site (Fig. 6B). Helix de pivots at its base with the Cα of L-Ser^209^ shifted 0.33 Å, while L-Val^221^ Cα is shifted 3.52 Å with no observable alterations in TMHs D and E, which are superimposable in the two structures (Fig. 6A). To our knowledge, this is the first structure of a closed Q_B_ site in a native RC, and comparison with the holo (Q_B_-bound) structure shows that conformational changes are required to admit quinone before its reduction. L-Phe^217^ rotates to form a π-stacking interaction with the quinone head group, and helix de shifts outward, allowing the backbone of L-Gly^222^ and side chain of L-Tyr^223^ to form a hydrogen bond network that stabilizes the holo structure (Fig. 6, A and C).

It is unexpected that the conformational changes observed in this study have not previously been reported, despite the availability of several structures of RCs with LH1 removed that lack Q_B_. These include Q_B_-depleted structures from *Blc. viridis* (PDB ID: 3PRC) (*27*), *Tch. tepidum* (PDB ID: 1EYS) (*28*), and *Rba. sphaeroides* (PDB ID: 1OGV) (*29*), all of which are near-identical to their respective holo Q_B_ structures. Close inspection of 3PRC reveals that an LDAO (Lauryldimethylamine oxide) detergent molecule is bound at the entrance to the Q_B_ site, which may prevent the rearrangement to the closed conformation. While no LDAO was resolved in the equivalent position in 1EYS or 1OGV, these RCs were prepared using the same detergent and may therefore be subject to the same effect. The crystal structure of the *Rba. sphaeroides* RC cocrystalized with cytochrome *c*_2_ (PDB ID: 1L9B) also appears to have a closed Q_B_ site. However, in this case, the N-terminal region of the RC-M polypeptide, which interacts with the Q_B_ binding site via an H-bond to the helix de Tyr residue, adopts a non-native conformation, and Q_B_ conformational changes were not explored further (*30*). Reassuringly, we see no such deformations to the M-polypeptide in the RC-LH1_14_-W structure, which is almost identical to the RC-LH1_16_ RC in its N-terminal region. It should also be noted that the apo RCs in the PDB were solved following detergent-based eradication of the LH1 antenna, which removes the internal quinone pool and lipids that reside in the gap between the RC and the inner face of the surrounding LH1 ring (*31*, *32*). The RC remains functional, because all of its cofactors are retained with the exception of the dissociable Q_B_ quinone, which is more labile and often lost during preparation (*33*). Furthermore, the removal of LH1 and native annular lipids from the RC is known to have consequent effects on functionality, such as lowered lifetime of the charge separated P^+^Q_B_^−^ state (*31*, *34*, *35*). We therefore speculate that the presence of a native LH1 ring surrounding the RC, preserving the local environment adjacent to Q_B_, may have facilitated the observation of the “closed” Q_B_ site.

Although the apo (no Q_B_ quinone) and holo structures represent only two snapshots of turnover at the Q_B_ site rather than a sequence of events, there is an indication that binding could be gated to prevent substrate inhibition by rebinding of quinol. A quinol approaching the apo Q_B_ site could interact differently to a quinone, resulting in its rejection by the RC. Conformational changes have long been suggested to play a role in the binding and reduction of quinone. RCs that have been frozen after dark adaptation are impaired in their ability to reduce quinone (*36*); x-ray crystallography has shown that this impairment is due to the Q_B_ quinone being trapped in a “distal” conformation ~4.5 Å from the active proximal position (*26*, *37*). We propose that this distal binding conformation is a snapshot of an intermediate state between the apo and holo structures that follows the initial interaction with quinone and opening of the Q_B_ site.

There is structural and functional conservation within the type II RCs found in some phototrophic bacteria and the PSII complexes of cyanobacteria, algae, and plants (*38*). The structural alignments shown in Fig. 6 (D and E) emphasize similarities between Q_B_ sites of PSII RCs and the bacterial RC complex; such comparisons have long served as models to study quinone binding and reduction in these closely related systems. Previous publications have suggested that conformational changes accompany quinone reduction by PSII (*39*, *40*). Thus, given the evolutionary conservation of RCs, this previously unobserved binding mechanism may also apply to the Q_B_ site of the PSII RCs in oxygenic phototrophs.

## MATERIALS AND METHODS

### Growth of *Rps. palustris* strains

The Δ*pufW* (unmarked deletion of *pufW*) and PufW-His (C-terminally 10× His-tagged protein-W expressed from the native *pufW* locus) strains of *Rps. palustris* CGA009 were described in our previous work (*16*). These strains and the isogenic wild-type parent were recovered from cryo-stocks [stored at −80°C in LB with 50% (w/v) glycerol] by streaking a small quantity of cells onto PYE (5 g liter^−1^ each of peptone, yeast extract, and succinate) agar [1.5% (w/v)] plates. Plates were incubated under anaerobic conditions overnight in the dark at room temperature and then illuminated with white light (~50 μmol m^−2^ s^−1^) provided by OSRAM 116-W halogen bulbs (RS Components, UK) for 3 to 5 days until single colonies appeared. A single colony was used to inoculate 10-ml M22+ medium (*41*) supplemented with 0.1% (w/v) casamino acids (from now on referred to as M22). This culture was grown under microoxic conditions in the dark at 34°C with shaking at 180 rpm for 48 hours and then used to inoculate a 70-ml culture grown under the same conditions for 24 hours. A volume of 1 ml of the semi-aerobic culture was used to inoculate 30 ml of M22 media in a screw-capped clear glass 30 ml universal and grown with illumination (~50 μmol m^−2^ s^−1^) for 48 hours with agitation via a sterile magnetic stir bar. The 30-ml culture was then used to inoculate ~1-liter cultures under the same conditions, which was subsequently used to inoculate ~9-liter cultures illuminated at ~200 μmol m^−2^ s^−1^ for 72 hours. Cells were harvested by centrifugation at 7132 RCF for 30 min, resuspended in ~10 ml of 20 mM tris-HCl (pH 8.0), and stored at −20°C until required.

### Preparation of solubilized *Rps. palustris* cellular membranes

After thawing, a few crystals of deoxyribonuclease I (Merck, UK), lysozyme (Merck, UK), and two Roche cOmplete Protease Inhibitor Cocktail tablets (Merck, UK) were added to the resuspended cells. Cells were broken by 8 to 12 passes in a French pressure cell (Aminco, USA) at 20,000 psi. Following removal of unbroken cells and insoluble debris by centrifugation at 18,500 RCF for 15 min at 4°C, membranes were pelleted from the deeply pigmented lysates by centrifugation at 113,000 RCF for 2 hours at 4°C. The soluble fraction was discarded, and the pigmented membranes were resuspended in 100 to 200 ml of 20 mM tris-HCl (pH 8.0) and homogenized until no visible aggregates remained. Resuspended membranes were solubilized by incubation in 20 mM tris-HCl (pH 8.0) containing 2% (w/v) β-DDM (Anatrace, USA) for 1 hour at 4°C in the dark with gentle stirring, followed by centrifugation at 150,000 RCF for 1 hour at 4°C to remove residual insoluble material.

### Purification of RC-LH1_16_ complexes

Solubilized membranes from the Δ*pufW* strain were applied to a 50-ml DEAE Sepharose ion-exchange column preequilibrated with three column volumes (CVs) of binding buffer [20 mM tris-HCl (pH 8.0) containing 0.03% (w/v) β-DDM]. The column was washed with two CVs of binding buffer and then with two CVs of binding buffer containing 50 mM NaCl. RC-LH1_16_ complexes were eluted over a linear gradient of 150 to 300 mM NaCl (in binding buffer) over 1.75 CVs, with residual bound complexes eluted over 0.5 CVs with binding buffer containing 300 mM NaCl. Absorption spectra were collected between 250 and 1000 nm, and fractions with a ratio of absorbance at 880 to 280 nm (A880/A280) above 1 were retained, diluted twofold in binding buffer, and purified on the DEAE column again using the same procedure. Fractions with A880/A280 ratios above 1.7 and A880/A805 ratios above 3.0 were diluted and subjected to a third round of ion-exchange, retaining fractions with A880/A280 ratios above 2.2 and A880/A805 ratios above 5.0. The partially purified complexes were concentrated to ~2 ml in Amicon 100,000 molecular weight cutoff (MWCO) centrifugal filters (Merck, UK), loaded onto a Superdex 200 16/600 size exclusion column (GE Healthcare, USA) preequilibrated with 1.5 CVs of binding buffer containing 200 mM NaCl, and then eluted over 1.5 CVs in the same buffer. Absorption spectra of the size exclusion fractions were collected, and those with A880/A280 ratios over 2.4 and A880/A805 over 5.8 were concentrated to an A880 of 100 and immediately used for cryo–transmission EM (cryo-TEM) grid preparation or stored at −80°C until required.

### Purification of RC-LH1_14_-W complexes

Solubilized membranes from the PufW-His strain were applied to a 20-ml HisPrep FF Ni-NTA Sepharose column (GE Healthcare) preequilibrated with IMAC buffer [20 mM tris-HCl (pH 8.0) containing 200 mM NaCl and 0.03% (w/v) β-DDM]. The column was washed with five CVs of IMAC buffer and then five CVs of IMAC buffer containing 10 mM histidine. Core complexes were eluted from the column with five CVs of IMAC buffer containing 100 mM histidine. Fractions containing RC-LH1_14_-W complexes were concentrated to ~10 ml in a stirred cell containing an Amicon 100,000 MWCO filter (Merck, UK), diluted 20-fold with binding buffer, and loaded onto a 25-ml DEAE sepharose column preequilibrated with four CVs of binding buffer. The column was washed with four CVs of binding buffer, and then complexes were eluted over a linear gradient from 0 to 100 mM NaCl (in binding buffer) over eight CVs, with residual complexes eluted over a further four CVs of binding buffer containing 100 mM NaCl. Fractions with an A880/A280 ratio above 2.4 and A880/A805 ratio above 4.6 were pooled, concentrated to ~2 ml in Amicon 100,000 MWCO centrifugal filters, loaded onto a Superdex 200 16/600 size exclusion column preequilibrated with 1.5 CVs of IMAC buffer, and then eluted over 1.5 CVs in the same buffer. Absorption spectra of the size exclusion fractions were collected, and those with A880/A280 ratios over 2.1 and A880/A805 over 4.6 were concentrated to an A880 of 100 and immediately used for cryo-TEM grid preparation or stored at −80°C until required.

### Cryo-TEM grid preparation

Cryo-TEM grids were prepared using a Leica EM GP plunge freezer. Complexes were diluted in IMAC buffer to an A880 of 50, and 5 μl was loaded onto freshly glow-discharged QUANTIFOIL 1.2/1.3 carbon-coated copper grids (Agar Scientific, UK). The grids were incubated for 30 s at 20°C and 60% relative humidity before blotting for 3 s and plunge-freezing in liquid ethane at −176°C.

### Cryo-TEM data collection

Data for the RC-LH1_14_-W complex were recorded at eBIC (Electron Bio-imaging Centre) (Diamond Light Source, UK) on a Titan Krios microscope operating at 300-kV accelerating voltage at a nominal magnification of 130,000 × with an energy-selecting slit of 20 eV. Data were collected in counting mode recording images with a Gatan 968 GIF Quantum with a K2 summit detector. The calibrated pixel size was 1.048 Å, and the dose rate was 3.83 e^−^ Å^−2^ s^−1^. Movies were collected over 11 s and dose-fractionated into 40 fractions. The microscope was refocused using a carbon-coated area before collecting three movies per hole. Overall, 3130 movies were collected with defocus values between −1 and −3 μm.

Data for the RC-LH1_16_ complex were collected at the Astbury Biostructure Laboratory (University of Leeds, UK) using an identical microscope. Data were collected in counting mode at a magnification of 130 k with a calibrated pixel size of 1.065 Å at a dose of 4.6 e^−^ Å^−2^ s^−1^. Movies were recorded over 12 s and dose-fractionated into 48 fractions. Overall, 3359 movies were collected with defocus values between −1 and −3 μm.

### Cryo-TEM data processing

All data processing was performed within the Relion 3.0 pipeline (*42*). Beam-induced motion correction was performed with dose weighting using Motioncorr 2 (*43*), followed by determination of CTF (contrast transfer function) parameters with CTFFIND 4.1 (*44*). Typical micrographs after these initial processing stages are shown in fig. S16. Autopicking templates were generated by manual picking of ~1000 particles with a box size of 250 pixels and reference-free two-dimensional (2D) classification, rejecting those classes that conform to sample contamination or had no discernible features. Autopicking was then performed on all micrographs resulting in 849,359 particles for the RC-LH1_14_-W and 476,547 particles for the RC-LH1_16_ complex. All picked particles were subjected to two rounds of reference-free 2D classification, and particles conforming to carbon areas, sample contamination, particles with no discernible features or strongly overlapping particles were rejected following each run, resulting in 772,033 (90.9%) and 359,678 (75.5%) particles being used for 3D classification for RC-LH1_14_-W and RC-LH1_16_, respectively. Initial 3D reference models were generated using the stochastic gradient descent method. Selected particles were subjected to 3D classification into four classes with the initial model as a reference. The particles within the largest class were subjected to 3D refinement using the model from this class as a reference followed by masking of solvent areas using an initial low-pass filter at 15 Å, adding a soft edge of 6 pixels, and postprocessing correcting for the modulation transfer function of the Gatan K2 summit detector. For the RC-LH1_14_-W dataset, this initial model was modified by removing strong density at the mask edge, which was disconnected from the core-complex density in UCSF Chimera. The resulting models (at 3.91 and 4.16 Å resolution for RC-LH1_14_-W and RC-LH1_16_, respectively) were used as a reference for a second round of 3D classification using particles grouping into initial 3D classes that did not contain strong overlap with neighbors or lack discernible structural features. Following the second round of 3D classification, the highest resolution classes were selected [one class of 377,703 particles (44.5%) for RC-LH1_14_-W and two classes with a combined total of 260,752 particles (54.7%) for RC-LH1_16_, which were identical when aligned after only initially differing by a small rotation]. Selected particles were reextracted in a 400-pixel box and refined by 3D refinement. A solvent mask was generated with an initial low-pass filter of 15 Å, map extension by 3 pixels, and a soft mask of 3 pixels. The resultant maps were further refined using per-particle CTF refinement, per-particle motion correction, and a second round of per-particle CTF refinement, with 3D refinement, solvent masking and postprocessing after each step. Using an FSC (Fourier shell correlation) cutoff at 0.143, the final models for RC-LH1_14_-W and RC-LH1_16_ were at resolutions of 2.65 and 2.80 Å, respectively. The FSC curves of the final models are shown in fig. S17.

### Model building

All protein sequences were downloaded from UniProtKB: LH1-β (PufB; UniProt ID: Q6N9L5); LH1-α (PufA; UniProtID: Q6N9L4); RC-L (PufL; UniProt ID: O83005); RC-M (PufM; UniProt ID: A0A4Z7); RC-H (PuhA; UniProt ID: A0A4Z9); protein-W (PufW; UniProt ID: Q6N1K3). A homology model of the RC was constructed using SWISS-MODEL (*45*) with protein sequences for RC-L, RC-M, and RC-H and a crystal structure of the *Rba. sphaeroides* RC as a template (PDB ID: 5LSE) (*46*). The resulting model was fit into the map using the “fit in map” tool within UCSF Chimera (*47*), the protein structure was refined, and cofactors [4× BChl *a* (monomer library residue name = BCL), 2× BPh *a* (BPH), one or two UQ_10_ (U10), one nonheme iron (Fe), and one 3,4-didehydrorhodopin (QAK)] were added using Coot (*48*). As QAK was not available in the monomer library, it was parameterized using the eLBOW tool in PHENIX (*49*).

Next, the LH1 subunits were constructed. Initially, the Autobuild tool in PHENIX (*49*) was used to automatically build part of the LH1 sequence using the map and the LH1-α and LH1-β protein sequences as inputs. The most complete LH1 subunit was selected, extracted, and loaded into Coot where missing sequence was manually added, and the entire structure was manually refined before addition of the two BChls *a* (BCL) and one spirilloxanthin (CRT) [assigned on the basis of the density and known carotenoid content of LH1 complexes of related *Rps.* species (*17*)]. The complete LH1 subunit was duplicated and docked into a neighboring nonmodeled region of LH1 density using the UCSF Chimera “dock in map tool” followed by refinement in Coot; this process was repeated until all LH1 subunits had been modeled. For the RC-LH1_14_-W structure, the remaining subunit (protein-W) was modeled by extracting the unassigned density in Coot, segmenting the protein from the remaining nonprotein components of the map in USCF Chimera and building an initial model with the Autobuild tool in PHENIX (*49*). Any missing sequence was added to the resulting model in Coot (*48*) followed by manual refinement of the entire subunit. The remaining unassigned density was fit with a combination of lipids (PDB monomer library IDs for CDL = CDL, POPC = 6PL, and POPG = PGT), β-DDM detergent (LMT), and UQ_10_ molecules (U10). The complete initial models were refined using cycles of PHENIX refine (*49*) and manual refinement in Coot (*48*) until the model statistics and visual quality of the fits could not be improved further. Last, local map sharpening was applied using LocScale (*50*), followed by several additional cycles of modeling unassigned density and automatic and manual refinement.

The individual peptides, cofactors, and additional lipids and quinones docked within their corresponding density are shown in figs. S18 to S23. Statistics for the final models are displayed in table S1.

### Ultraviolet/Vis/NIR absorption spectroscopy

Ultraviolet/visible/near-infrared absorption spectra were collected on a Cary60 spectrophotometer (Agilent, USA) scanning between 250 and 1000 nm at 1-nm intervals with a 0.1-s integration time, unless otherwise stated.

### Circular dichroism spectroscopy

Samples were diluted to an A880 of 1 in a 2-mm path quartz cuvette, and absorption spectra were collected between 400 and 1000 nm. Circular dichroism spectra were collected on a Jasco 810 spectropolarimeter (Jasco, Japan) between 400 and 950 nm at 1-nm intervals at a scan rate of 20 nm min^−1^.

### Determination of molar extinction coefficients

Molar extinction coefficients were determined by diluting core complexes to an A880 of ~50. A 10-μl volume was diluted in 990-μl binding buffer or methanol, and absorption spectra were collected immediately to minimize BChl degradation. Extinction coefficients were determined by calculating the BChl content of each methanol sample using an extinction coefficient of 54.8 mM^−1^ cm^−1^ at 771 nm (*51*). The measured BChl concentration was divided by 32 (RC-LH1_14_-W) or 36 (RC-LH1_16_) to determine the core complex concentration, which was subsequently used to determine an extinction coefficient from the absorption spectra of the same samples in buffer collected in parallel. Each sample was measured in triplicate, and the average absorbance for the BChl Q_y_ maxima was used for the calculations. The extinction coefficients determined were 3280 ± 140 mM^−1^ cm^−1^ at 878 nm for RC-LH1_14_-W and 3800 ± 30 mM^−1^ cm^−1^ at 880 nm for RC-LH1_16_.

### Quantification of UQ_10_

UQ_10_ was quantified on the basis of the method in (*52*). Briefly, reverse-phase HPLC (RP-HPLC) was performed using an Agilent 1200 HPLC system. Approximately 0.02 nmol of RC-LH1_16_ or RC-LH1_14_-W was dissolved in 50 μl of 50:50 methanol:chloroform containing 0.02% (w/v) ferric chloride and injected onto a Beckman Coulter Ultrasphere ODS 4.6 mm × 25 cm column preequilibrated in HPLC solvent (80:20 methanol:2-propanol) at 40°C at 1 ml^−1^ min^−1^. Isocratic elution was performed in HPLC solvent for 1-hour monitoring absorbance at 275 nm (UQ_10_), 450 nm (carotenoid), and 780 nm (BChl). The peak in the 275-nm chromatogram at 25.5 min, which did not contain any other detectable compounds, was integrated. The integrated area was used to calculate the molar quantity of extracted UQ_10_ by reference to a calibration curve calculated from injection of 0 to 5.8 nmol of pure standard (fig. S14). Each sample was analyzed in triplicate with reported errors corresponding to SD of the mean.

### Cytochrome *c* oxidation assays

Solutions containing RC-LH1 complexes at a maximum Q_y_ absorption of 0.1 were prepared with 30 μM reduced horse-heart cytochrome *c*_2_ (Merck, UK) and 0 to 50 μM UQ_2_ (Merck, UK). Three 1-ml samples were prepared at each UQ_2_ concentration and incubated at 4°C in the dark overnight to ensure they were fully dark-adapted before measurement. Solutions were loaded into an OLIS RSM1000 modular spectrophotometer equipped with a 300-nm blaze/500-line grating and a 1.24-mm entrance, 0.12-mm intermediate, and 0.6-mm exit slit. The sample and reference photomultiplier tubes had 600-nm long-pass filters placed at their entrances to exclude excitation light. Absorbance was monitored at 550 nm with an integration time of 0.15 s. Excitation light was delivered via a fiber optic cable from an 880-nm M880F2 LED (Light-emitting diode) (Thorlabs Ltd., UK) driven at 90% intensity using a DC2200 controller (Thorlabs Ltd., UK) at 90° to the measurement beam with a mirror opposite to return any light that was not initially absorbed by the sample. Absorbance was monitored for 10 s before illumination for 50 s. The absorbance was then monitored for a further 60 s in darkness to assess the extent of the spontaneous reduction of cytochrome *c*_2_^3+^ by quinol (see fig. S8 for raw data).

Data were processed by fitting of the linear initial rate over 0.5 to 10 s (dependent on UQ_2_ concentration) and averaging the rates of all three samples at each UQ_2_ concentration. Rates were converted into catalytic efficiencies using RC-LH1 concentrations calculated with their respective extinction coefficients, plotted in Origin pro 2019 (OriginLab, USA), and fit to the Michaelis-Menten model to determine apparent *K*_m_ and *K*_cat_ values.

### Transient absorption spectroscopy

For transient absorption measurements, RC-LH1 samples were diluted to ~2 μM in IMAC buffer containing 50 mM sodium ascorbate (Merck, USA) and 0.4 mM terbutryn (Merck, USA). The ascorbate was used as a sacrificial electron donor, while the terbutryn acts as a Q_B_ inhibitor to ensure that the RC primary donor remains in the reduced (i.e., not photooxidized) state throughout the measurements. Approximately 3 ml of sample was added to a 2-mm path length custom-built spinning cell (~0.1 m in diameter, 350 RPM) to ensure that the sample in the laser path has sufficient time to dark-adapt between excitation pulses. Samples were excited at 880 nm using ~100-fs laser pulses at a repetition rate of at 1 kHz (20 nJ for NIR or 100 nJ for Vis) provided by an amplified Ti:Sapphire laser system (Spectra Physics, USA). The samples were exposed to excitation light for a duration of ~30 min before data collection, the exposure results in Q_A_ inactivation (possibly by singly or doubly reducing Q_A_). Note, however, that this process is reversible, because the RCs slowly return to the Q_A_ active state following prolonged dark adaption. Transient spectra were measured using a Helios spectrometer (Ultrafast Systems, USA) with delay times from −10 to 7000 ps. The datasets were dechirped using Surface Xplorer software (Ultrafast Systems, USA), then combined, and normalized. The combined datasets were used to obtain decay-associated difference spectra using the CarpetView software package (Light Conversion Ltd., Lithuania) or fit to single-wavelength spectral evolutions in Origin using functions that consist of multiple exponentials convoluted with the instrument response (OriginLab, USA).

Photosynthetic membranes containing the LH1 complex lacking both the RC and peripheral LH2 antenna were prepared as described previously (*53*). The membranes were diluted in 20 mM tris (pH 8.0) and loaded into a 2-mm path length quartz cuvette. The sample was excited at 540 nm using 30-nJ laser pulses with delay times from −10 to 7000 ps. Datasets were processed as described for the *Rps. palustris* samples.

### Mass spectrometry

Membranes were pelleted by centrifugation at 150,000 RCF for 2 hours at 4°C and resuspended at an absorbance of 100 at 880 nm in 20 mM tris-HCl (pH 8.0) and 200 mM NaCl. Membranes were solubilized by gentle stirring in 2% (w/v) β-DDM for 1 hour at 4°C in the dark. Samples were diluted to a protein concentration of 2.5 mg ml^−1^ (Bio-Rad assay) in 100 mM triethylammonium bicarbonate (pH 8.0) (TEAB; Merck, UK). Further processing was adapted from a previously published method (*54*) starting with the dilution of 50 μg protein in a total of 50 μl of TEAB containing 1% (w/v) sodium laurate (Merck, UK). After sonication for 60 s, reduction with 5 mM tris(2-carboxyethyl)phosphine (Merck, UK) was carried out at 37°C for 30 min. For S-alkylation, the samples were incubated with 10 mM *S*-methyl methanethiosulfonate (Merck, UK), added from a stock 200 mM solution in isopropanol, at room temperature for 10 min. Proteolytic digestion was carried by the addition of 2 μg of trypsin/endoproteinase Lys-C mix (Promega, UK) and incubation at 37°C for 3 hours. The laurate surfactant was extracted by the addition of 50 μl of ethyl acetate and 10 μl of 10% (v/v) LC grade trifluoroacetic acid (TFA; Thermo Fisher Scientific, UK) with vortexing for 60 s. Phase separation was facilitated by centrifugation at 15,700 RCF for 5 min. The peptide-containing lower phase was carefully aspirated and desalted using a C_18_ spin column (Thermo Fisher Scientific, UK) according to the manufacturer’s protocol. After drying by vacuum centrifugation, the samples were dissolved in 0.5% TFA and 3% acetonitrile, and 500 ng was analyzed by nano-flow RP chromatography coupled to mass spectrometry using system parameters detailed previously (*55*).

Protein identification and quantification were carried out using MaxQuant v.1.5.3.30 (*56*) to search a *Rps. palustris* proteome database (www.uniprot.org/proteomes/UP000001426). The mass spectrometry proteomics data have been deposited to the ProteomeXchange Consortium via the PRIDE partner repository (http://proteomecentral.proteomexchange.org) with the dataset identifier PXD020402.

For analysis by RPLC coupled to electrospray ionization mass spectrometry, RC-LH1 complexes were prepared from wild-type *Rps. palustris* cells using a previously published method (*16*) to give a protein concentration of 2 mg ml^−1^ (Bio-Rad assay) in 20 mM Hepes (pH 7.8), 100 mM NaCl, and 0.03% (w/v) β-DDM. Ten micrograms of protein was extracted by precipitation using a 2D clean-up kit (GE Healthcare, USA) according to the manufacturer’s protocol, and the pellet was dissolved in 20 μl 60% (v/v) formic acid (FA), 20% (v/v) acetonitrile, and 20% (v/v) water. Five microliters was analyzed by RPLC (Dionex RSLC) coupled to mass spectrometry (Maxis UHR-TOF, Bruker). Separation was performed using a MabPac 1.2 × 100 mm column (Thermo Fisher Scientific, UK) at 60°C and 100 μl min ^−1^ with a gradient of 85% (v/v) solvent A [0.1% (v/v) FA and 0.02% (v/v) TFA in water] to 85% (v/v) solvent B [0.1% (v/v) FA and 0.02% (v/v) TFA in 90% (v/v) acetonitrile] over 60 min. The mass spectrometer acquired 100 to 2750 *m/z* (mass charge ratio) using a standard electrospray ionization source and default parameters. Mass spectra were mapped to subunits belonging to the complex with the aid of the ExPASy Bioinformatics Resource Portal FindPept tool (https://web.expasy.org/findpept/).

### Immunodetection of protein-W

Cells were grown under low (10 μM m^−2^ s^−1^), medium (30 μM m^−2^ s^−1^), or high (300 μM m^−2^ s^−1^) illumination for 72 hours in 100 ml of NF-M22 medium (M22 medium where the ammonium sulphate is omitted, and sodium succinate is replaced with sodium acetate) (*23*) in 100-ml screw-cap bottles. Cells were lysed by bead beating in 1:1 volumetric ratio with 0.1 μm glass beads over five 30-s cycles, cooling on ice for 5 min in between. Insoluble material, unbroken cells, and glass beads were removed by centrifugation at 16,000 RCF for 10 min in a benchtop microcentrifuge. Membranes were isolated on 40/15% (w/w) sucrose gradients in 20 mM tris-HCl (pH 8.0) in a Ti 70.1 rotor at 100,000 RCF for 10 hours.

Immunodetection of the His-tag on PufW was performed as described in our previous work (*16*). Briefly, purified core complex (11.8 nM) or membranes containing the same concentration of RC (as determined by oxidized minus reduced difference spectra and matched loading on stained gels) were diluted twofold in 2× SDS-loading buffer (Merck, UK), and proteins were separated on replica 12% bis-tris NuPage gels (Thermo Fisher Scientific, UK). One gel was stained with Coomassie Brilliant Blue (Bio-Rad, UK) to access loading and visualize the RC-L subunit. Protein on the second gel was transferred to methanol-activated polyvinylidene fluoride (PVDF) membranes (Thermo Fisher Scientific, UK) for immunodetection. PVDF membranes were blocked in 50 mM tris-HCl (pH 7.6), 150 mM NaCl, 0.2% (v/v) Tween-20, and 5% (w/v) skimmed milk powder before incubation with anti-His primary antibody (A190-114A, Bethyl Laboratories, USA) diluted 1:1000 in antibody buffer [50 mM tris-HCl (pH 7.6), 150 mM NaCl, and 0.05% (v/v) Tween-20] for 4 hours. After three 5-min washes in antibody buffer, the membrane was incubated with anti-mouse secondary antibody (diluted 1:10,000 in antibody buffer) conjugated with horseradish peroxidase (Sigma-Aldrich, UK) to allow (after three 5-min washes in antibody buffer) detection using the WESTAR ETA C 2.0 chemiluminescent substrate (Cyanagen, Italy) and an Amersham Imager 600 (GE Healthcare, UK).

Images were processed in ImageJ (*57*) by plotting intensity profiles for each stained gel or immunoassay lane, integrating the areas under the peaks and calculating intensity ratios for the RC-L (stained gel) and protein-W (immunoassay). These ratios were converted into molar ratios by assuming the RC-L to protein-W ratio in the pure RC-LH1_14_-W sample to be 1:1 and normalizing the entire dataset accordingly.

## Supplementary Material

http://advances.sciencemag.org/cgi/content/full/7/3/eabe2631/DC1

Adobe PDF - abe2631_SM.pdf

Structures of Rhodopseudomonas palustris RC-LH1 complexes with open or closed quinone channels

## SUPPLEMENTARY MATERIALS

Supplementary material for this article is available at http://advances.sciencemag.org/cgi/content/full/7/3/eabe2631/DC1

## Appendix

View/request a protocol for this paper from *Bio-protocol*.
