# Supplementary material for: Structures of Rhodopseudomonas palustris RC-LH1 complexes with open or closed quinone channels
Source: Sci Adv. 2021 Jan 13;7(3):eabe2631. doi: 10.1126/sciadv.abe2631 (PMC7806223; doi:10.1126/sciadv.abe2631)
Supplement: http://advances.sciencemag.org/cgi/content/full/7/3/eabe2631/DC1 [file supp_7_3_eabe2631__index.html]

Science Advances | Science AdvancesAAASSearchScience AdvancesMenu

## Supplementary Materials

# Structures of *Rhodopseudomonas palustris* RC-LH1 complexes with open or closed quinone channels

David J. K. Swainsbury, Pu Qian, Philip J. Jackson, Kaitlyn M. Faries, Dariusz M. Niedzwiedzki, Elizabeth C. Martin, David A. Farmer, Lorna A. Malone, Rebecca F. Thompson, Neil A. Ranson, Daniel P. Canniffe, Mark J. Dickman, Dewey Holten, Christine Kirmaier, Andrew Hitchcock, C. Neil Hunter

Download Supplement

**This PDF file includes:**

- Figs. S1 to S23
- Supplementary Text
- Tables S1 to S3
- References

**Files in this Data Supplement:**

- Adobe PDF - abe2631\_SM.pdf
