# Supplementary material for: Structures of Rhodopseudomonas palustris RC-LH1 complexes with open or closed quinone channels
Source: Sci Adv. 2021 Jan 13;7(3):eabe2631. doi: 10.1126/sciadv.abe2631 (PMC7806223; doi:10.1126/sciadv.abe2631)
Supplement: http://advances.sciencemag.org/cgi/content/full/7/3/eabe2631/DC1 [file supp_7_3_eabe2631__1.pdf]

Science Advances
AAAS

advances.sciencemag.org/cgi/content/full/7/3/eabe2631/DC1

Supplementary Materials for

Structures of *Rhodopseudomonas palustris* RC-LH1 complexes with open or closed quinone channels

David J. K. Swainsbury*, Pu Qian, Philip J. Jackson, Kaitlyn M. Faries, Dariusz M. Niedzwiedzki, Elizabeth C. Martin, David A. Farmer, Lorna A. Malone, Rebecca F. Thompson, Neil A. Ranson, Daniel P. Canniffe, Mark J. Dickman, Dewey Holten, Christine Kirmaier, Andrew Hitchcock, C. Neil Hunter*

*Corresponding author. Email: d.swainsbury@sheffield.ac.uk (D.J.K.S.); c.n.hunter@sheffield.ac.uk (C.N.H.)

Published 13 January 2021, *Sci. Adv.* **7**, eabe2631 (2021)
DOI: 10.1126/sciadv.abe2631

This PDF file includes:

Figs. S1 to S23
Supplementary Text
Tables S1 to S3
References

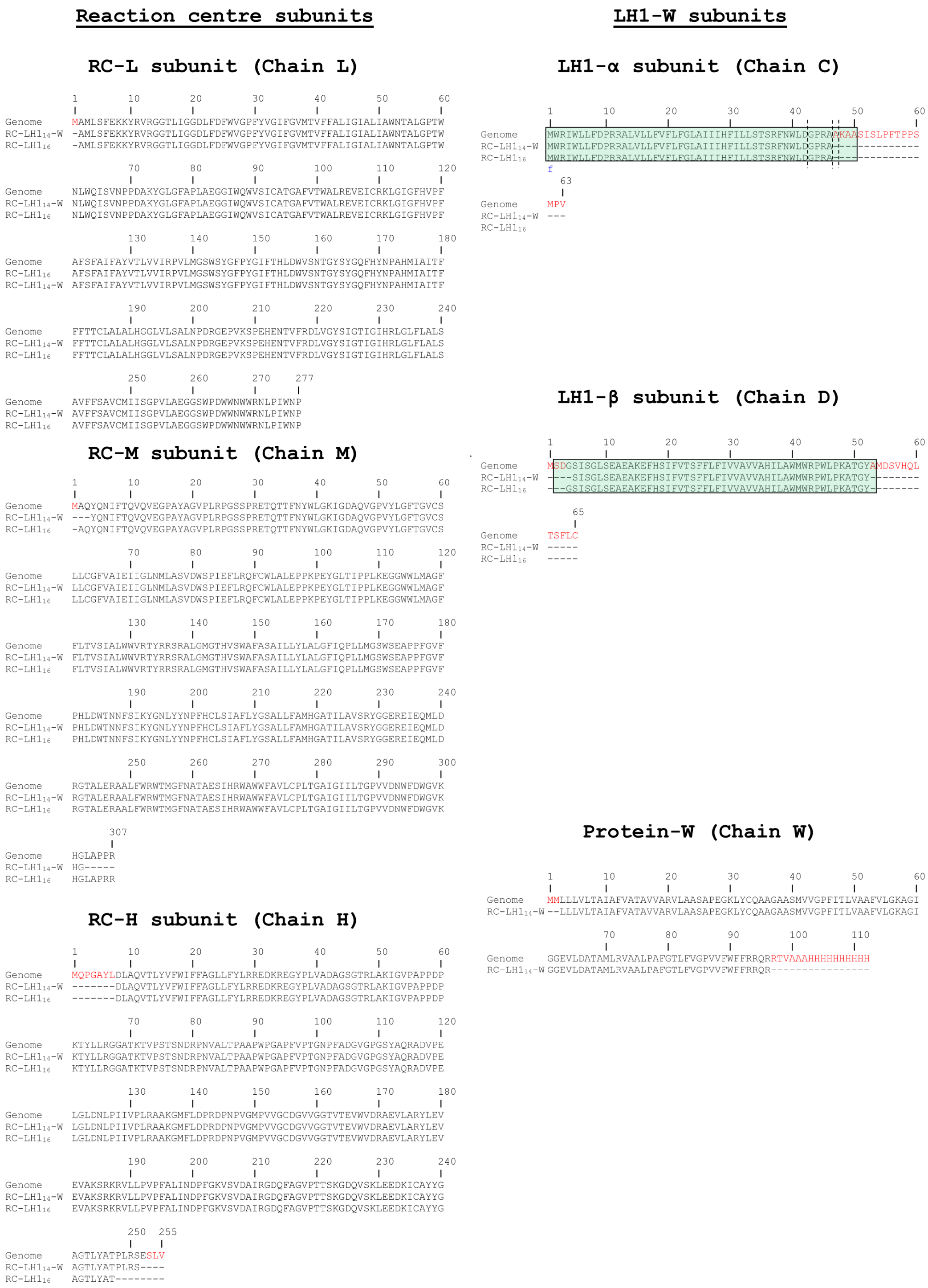

Fig. S1. Alignments of RC-LH1 core complex subunit amino acid sequences showing the resolved residues in the two RC-LH1 complexes. Residues that were not resolved in the structures are colored red in the sequences generated from the genome sequence (labelled genome). Structural sequences for LH1 α and β were taken from chains with the greatest level of coverage. Green boxes highlight α and β sequences observed by mass spectrometry with dashed lines showing alternate cleavage sites. The ‘f’ below the sequence indicates that Met1 is N-formylated.

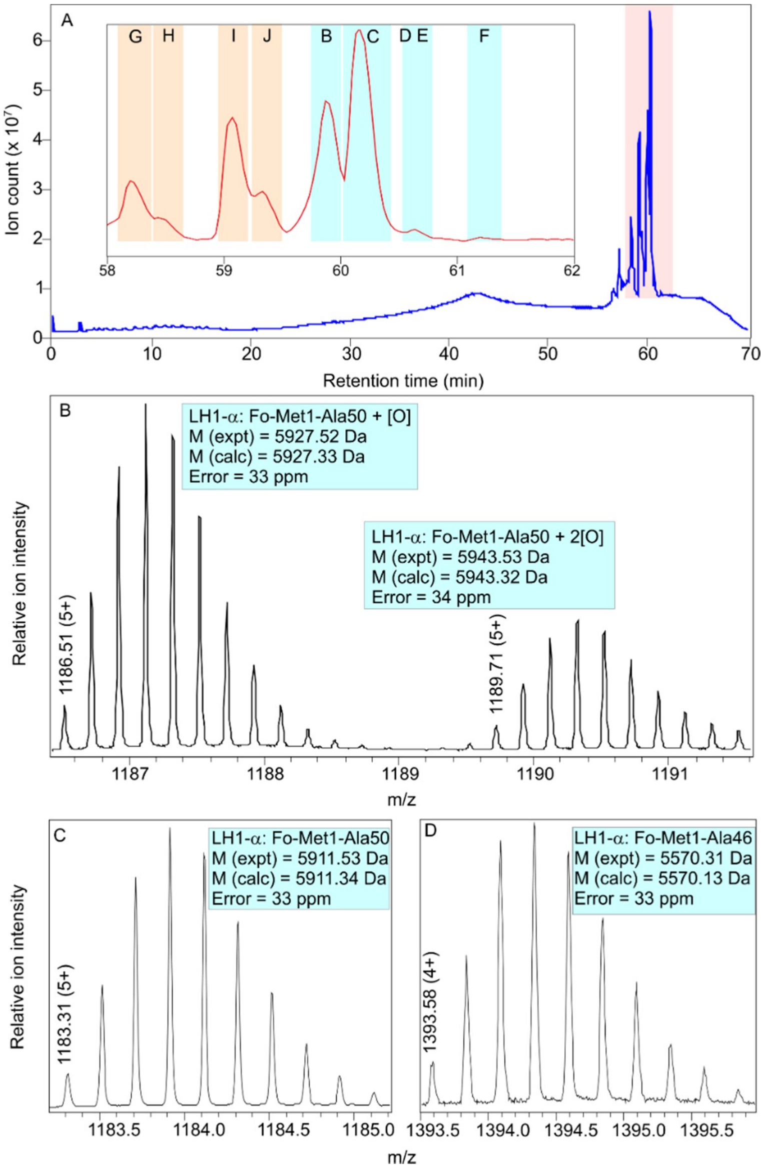
A
Ion count (x 10^7)
Retention time (min)
G
H
I
J
B
C
D E
F
B
LH1-α: Fo-Met1-Ala50 + [O]
M (expt) = 5927.52 Da
M (calc) = 5927.33 Da
Error = 33 ppm
LH1-α: Fo-Met1-Ala50 + 2[O]
M (expt) = 5943.53 Da
M (calc) = 5943.32 Da
Error = 34 ppm
1186.51 (5+)
1189.71 (5+)
Relative ion intensity
m/z
C
LH1-α: Fo-Met1-Ala50
M (expt) = 5911.53 Da
M (calc) = 5911.34 Da
Error = 33 ppm
1183.31 (5+)
D
LH1-α: Fo-Met1-Ala46
M (expt) = 5570.31 Da
M (calc) = 5570.13 Da
Error = 33 ppm
1393.58 (4+)
Relative ion intensity
m/z

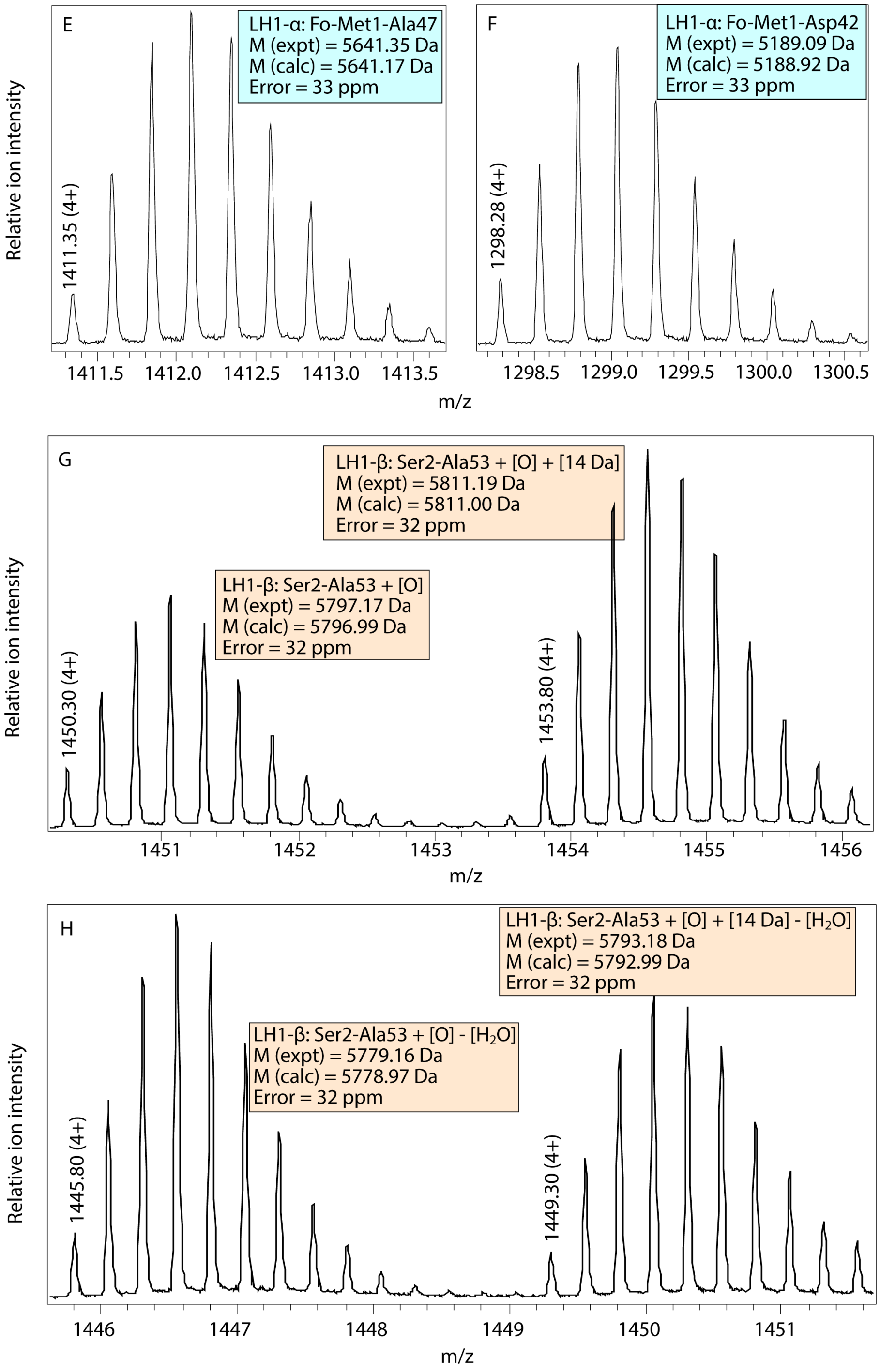

E
LH1-α: Fo-Met1-Ala47
M (expt) = 5641.35 Da
M (calc) = 5641.17 Da
Error = 33 ppm
1411.35 (4+)
Relative ion intensity
1411.5
1412.0
1412.5
1413.0
1413.5
F
LH1-α: Fo-Met1-Asp42
M (expt) = 5189.09 Da
M (calc) = 5188.92 Da
Error = 33 ppm
1298.28 (4+)
1298.5
1299.0
1299.5
1300.0
1300.5
m/z
G
LH1-β: Ser2-Ala53 + [O] + [14 Da]
M (expt) = 5811.19 Da
M (calc) = 5811.00 Da
Error = 32 ppm
LH1-β: Ser2-Ala53 + [O]
M (expt) = 5797.17 Da
M (calc) = 5796.99 Da
Error = 32 ppm
1450.30 (4+)
1453.80 (4+)
Relative ion intensity
1451
1452
1453
1454
1455
1456
m/z
H
LH1-β: Ser2-Ala53 + [O] + [14 Da] - [H2O]
M (expt) = 5793.18 Da
M (calc) = 5792.99 Da
Error = 32 ppm
LH1-β: Ser2-Ala53 + [O] - [H2O]
M (expt) = 5779.16 Da
M (calc) = 5778.97 Da
Error = 32 ppm
1445.80 (4+)
1449.30 (4+)
Relative ion intensity
1446
1447
1448
1449
1450
1451
m/z

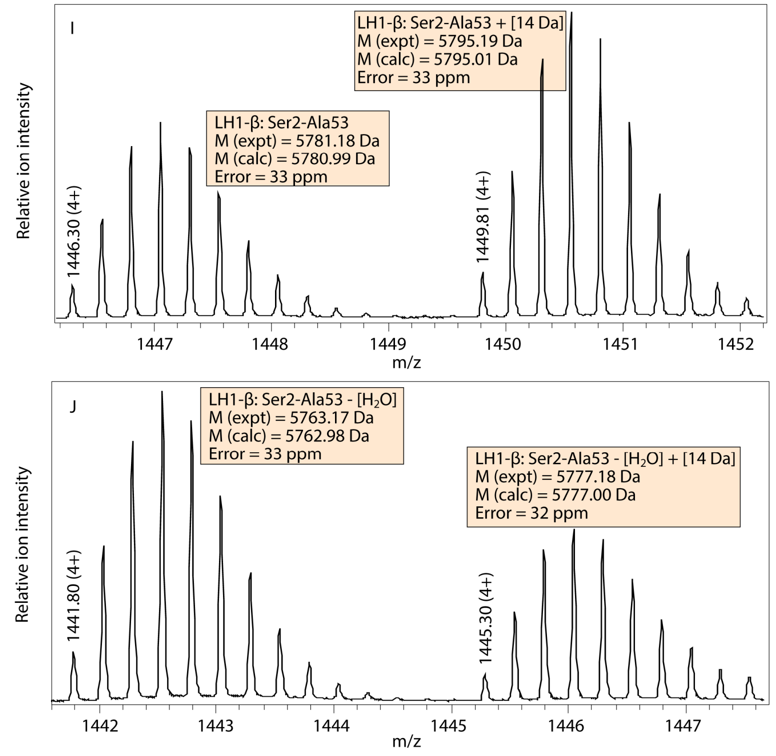

Fig. S2. Intact mass analysis of the purified *Rps. palustris* RC-LH1 using RPLC-ESI-MS. Panel A shows the total ion chromatogram with the retention time range for the LH1 polypeptides (58–62 min) highlighted in red and shown on an expanded scale in the inset panel. Low level spectral peaks at 56-58 min that could not be accurately deconvoluted were tentatively mapped at >27 kDa to RC subunits (not shown). Ions potentially relating to protein-W were below the limit of detection, presumably due to its low abundance at native ratios to the LH1 polypeptides. Peaks mapping to LH1-α and LH1-β are highlighted in blue and orange, respectively, with the panels displaying mass spectral evidence indicated. In all cases, the isotopomer series ions shown are the highest intensity charge states that occurred in the spectra with the monoisotopic ions labelled with their m/z values. Deconvolution reveals that their zero charge monoisotopic masses provide strong evidence that LH1-α is not N-terminally processed by either N-formylase or methionine aminopeptidase while LH1-β undergoes Met1 excision. However, both are C-terminally truncated, presumably *in vivo*. There were no ions detected in any of the spectra to indicate the occurrence of full length gene products. The monoisotopic masses were consistent with the identification of: (B) Mono- and di-oxidized derivatives of LH1-α (formyl-Met1-Ala50), formed by reaction with atmospheric oxygen. (C) LH1-α (formyl-Met1-Ala50). (D) LH1-α (formyl-Met1-Ala46). (E) LH1- α (formyl-Met1-Ala47). (F) LH1-α (formyl-Met1-Asp42). (G) Mono-oxidized derivatives of LH1-β (Ser2-Ala53 and Ser2-Ala53[Δ+14Da]). (H) As per (G) with elimination of water, most probably resulting from gas phase reactions involving Ser or Glu side-chains in the ionization source. (I) LH1-β (Ser2-Ala53 and Ser2-Ala53[Δ+14Da]). (J) As per (I) with elimination of water. The evidence for a Δ+14 Da form of LH1-β (Ser2-Ala53) suggests the possibility of either N- or O-methylation, however the location of this modification could not be determined.

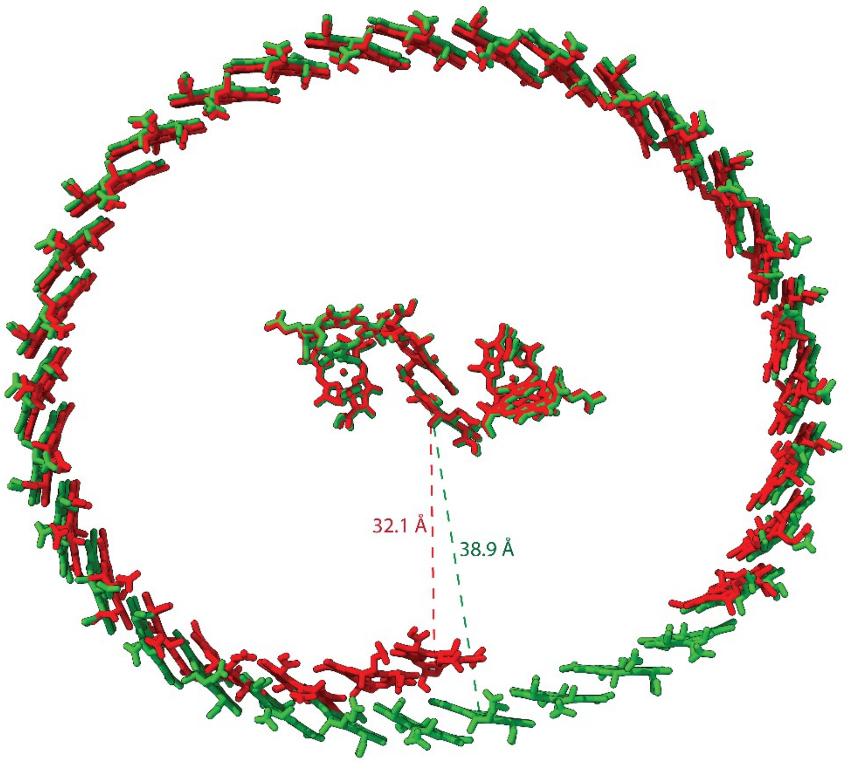

Fig. S3. Overlaid structures of the LH1 and RC BChl and RC BPh macrocycles for RC-LH1$_{16}$ (green) and RC-LH1$_{14}$-W (red). Distances between the magnesium atoms for the LH1 BChls closest to the RC primary donor are shown with labelled dashed lines.

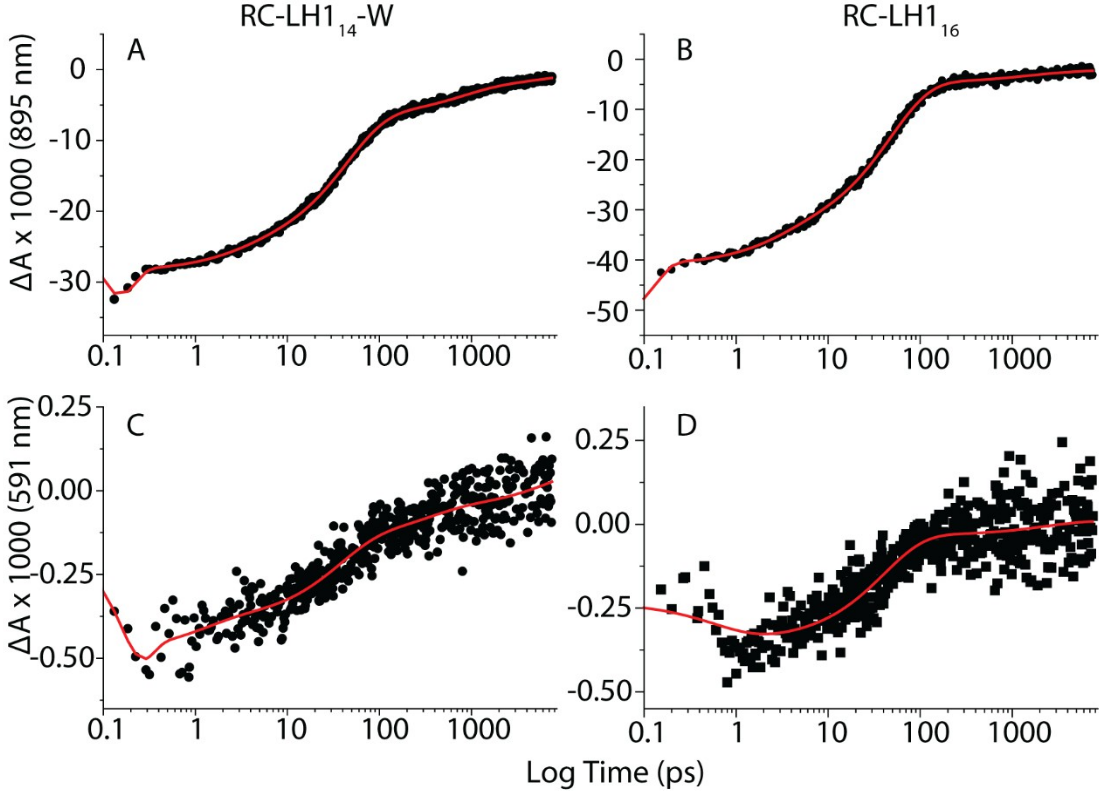

Fig. S4. Picosecond spectral changes following excitation of RC-LH1$_{14}$-W and RC-LH1$_{16}$ at 880 nm. Spectral changes for RC-LH1$_{14}$-W (panels A, C) and RC-LH1$_{16}$ (panels B, D) at 895 (panels A, B; the Q_y bleaching for the LH1 BChls) and 591 nm (panels C, D; the Q_x bleaching for the LH1 BChls) following excitation with a 100 fs laser flash at 880 nm. Raw data (black circles) were fit to a multi-exponential model (red lines). The fitted values are shown in Table S2.

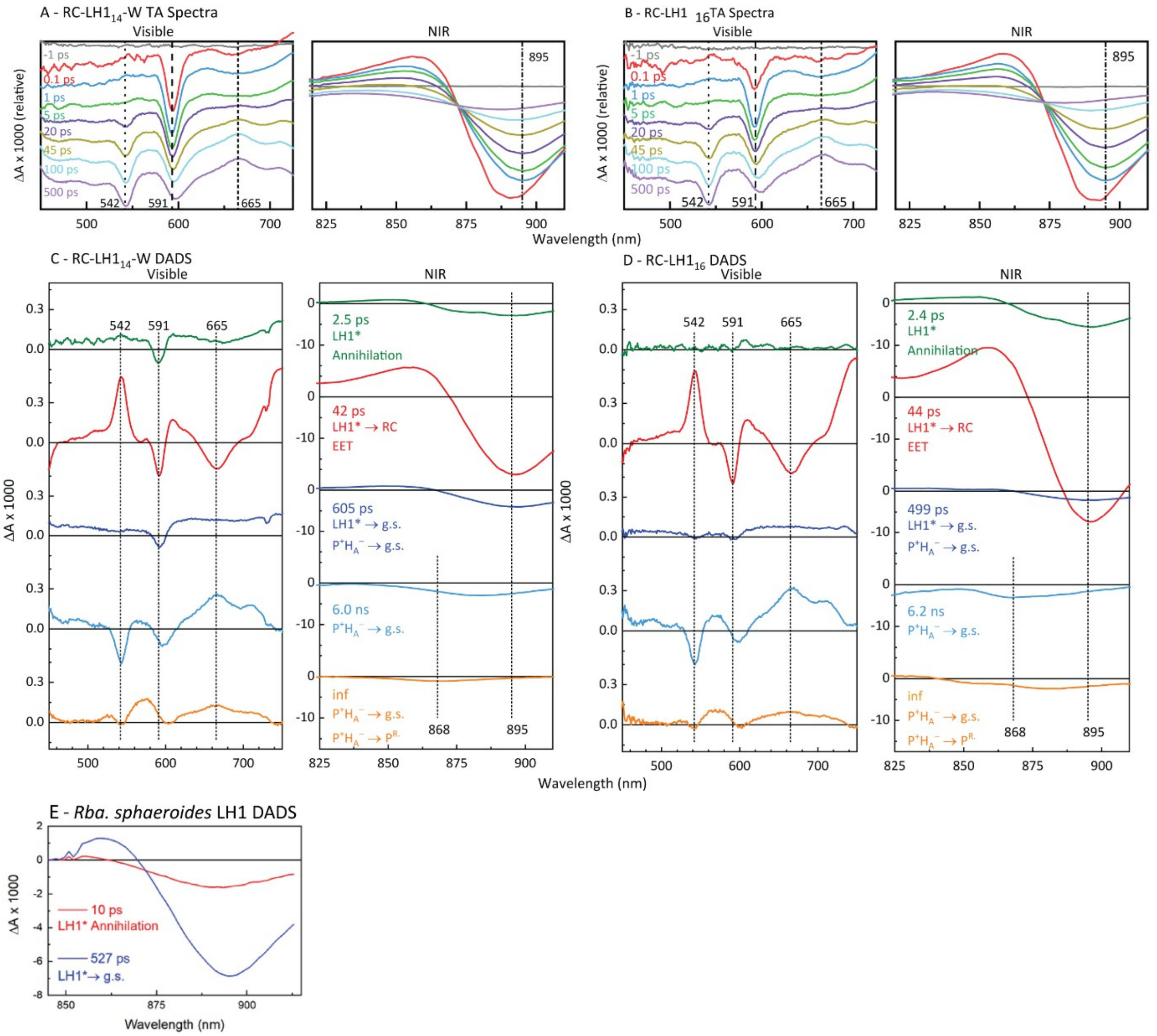

Fig. S5. Transient absorption (TA) spectra and decay associated difference spectra (DADS) for the RC-LH1$_{14}$-W and RC-LH1$_{16}$ complexes. Panels A and B show TA spectra for RC-LH1$_{14}$-W and RC-LH1$_{16}$, respectively, in the visible and near-infrared (NIR) spectral regions. Panels C and D show DADS in the visible and NIR spectral regions for the RC-LH1$_{14}$-W and RC-LH1$_{16}$ core complexes, respectively. Global analysis was performed to deconvolute the time-resolved spectra in Carpetview. The visible and NIR spectra were combined (Fig. 2C-D) and modelled assuming parallel decay of temporally distinct non-reversible components. The resultant DADS are labelled with their associated process and time constants (inf being slower than the 7 ns measurement window time), assigned based upon known spectral signatures elucidated in previous studies (*59*, *60*). LH1* = singlet excited state of the LH1 BChls, RC = RC primary donor, EET = excitation energy transfer, g.s. = ground state, $P^+H_A^-$ = RC charge separated state, P^R = RC primary donor triplet state. Panel E shows DADS of LH1 from *Rba. sphaeroides* lacking the RC (thus lacking the LH1→RC EET and all RC processes), which was used as a reference for assignment of the LH1* annihilation and LH1*→GS signals in the two *Rps. palustris* samples (preparation of the *Rba. sphaeroides* LH1 is detailed in ref (*53*))

Supplementary text

The DADS in Figure S5 show the competing processes/states present in the RC-LH1 samples following excitation. The process of particular interest to this work is LH1→RC energy transfer (EET, ~42 ps, red lines) and subsequent charge recombination following electron transfer (ET) within the RC (~5-6 ns, light blue lines). Here, LH1→RC EET (~42 ps, red lines) is the first step in a two-step process that involves EET from the excited LH1 (LH1*) to the RC to make the RC primary electron donor (P*), followed by rapid (3-4 ps) $P^* \rightarrow P^+H_A^-$ ET within the RC. The spectral signatures in the DADS are decay of LH1 bleaching at 896 nm (due to $LH1^* \rightarrow P^*$ EET) and the growing in of $P^+H_A^-$ features, namely the bleaching of the H_AQ_x band at 542 nm and the formation of the H_A^- anion band at 665 nm (due to $P^* \rightarrow P^+H_A^-$ ET). The subsequent decay of these $P^+H_A^-$ features (via $P^+H_A^-$ charge recombination) are clear in the ~6 ns spectra, as well as the infinity spectra (inf, orange lines) where some population of $P^+H_A^-$ decays to form the P triplet state (P^R) as well as ground state (g.s.) with a time constant greater than the timescale of the experiment. Note that it is known that $P^+H_A^-$ charge recombination is multi-exponential with at least three time constants spanning <1 ns to >10 ns; thus, the 6 ns observed here is an average. The other competing processes are as follows:

(1) The ~2.5 ps spectra (green lines) indicate excited state LH1 (LH1*) intra-ring annihilation (resulting from two excited states within the same LH1 ring), as seen from the LH1 decay of BChl Q_x bleaching (591 nm) and Q_y bleaching (~895 nm).

(2) As described in the Materials and Methods Transient Absorption Spectroscopy section, a high concentration of ascorbate (~10,000x the RC concentration) is added to the RC-LH1 samples to ensure that P is reduced (i.e., not in the photooxidized state) prior to each excitation flash (preventing the RCs from becoming "closed" and thus unable to participate in LH1→RC EET). However, a small fraction of RC-LH1 complexes (~5-15%) exhibit excited state LH1*→g.s. decay (500-600 ps, dark blue lines), probably due to some RCs remaining oxidized when exposed to the excitation pulse or the RC being absent from a small fraction of the LH1 complexes (likely due to damage during sample preparation). The LH1* excited state lifetime in the absence of the RC (i.e. deactivation of LH1* to the g.s.) in *Rba. sphaeroides* is ~500 ps (shown in figure S5E), which is in close agreement with the ~500-600 ps component found in the *Rps. palustris* RC-LH1 samples.

(3) The $P^+H_A^-$ state should primarily decay by well-known charge recombination pathways to produce the g.s. or P^R (*59*, *60*) (~6 ns and inf spectra, dark blue and orange lines respectively); however, a small fraction of the RC-$LH1_{16}$ sample still has active Q_A and exhibits $P^+H_A^- \rightarrow P^+Q_A^-$ ET (e.g., 542 nm bleaching decay). This is despite the RC Q_B being replaced by terbutryn (which cannot participate in electron transfer) and Q_A being predominantly inactivated by pre-exposure of the sample to the excitation laser (see materials and methods). $P^+H_A \rightarrow P^+Q_A^-$ ET typically has a time constant of ~200 ps (*59*, *60*), but the presence of this process in such a small fraction (~10%) of the sample and close overlap in time with LH1-only decay (~500 ps time constant) prevents the two states/processes from being resolved in the DADS and therefore are combined in the ~500 ps spectrum (dark blue line).

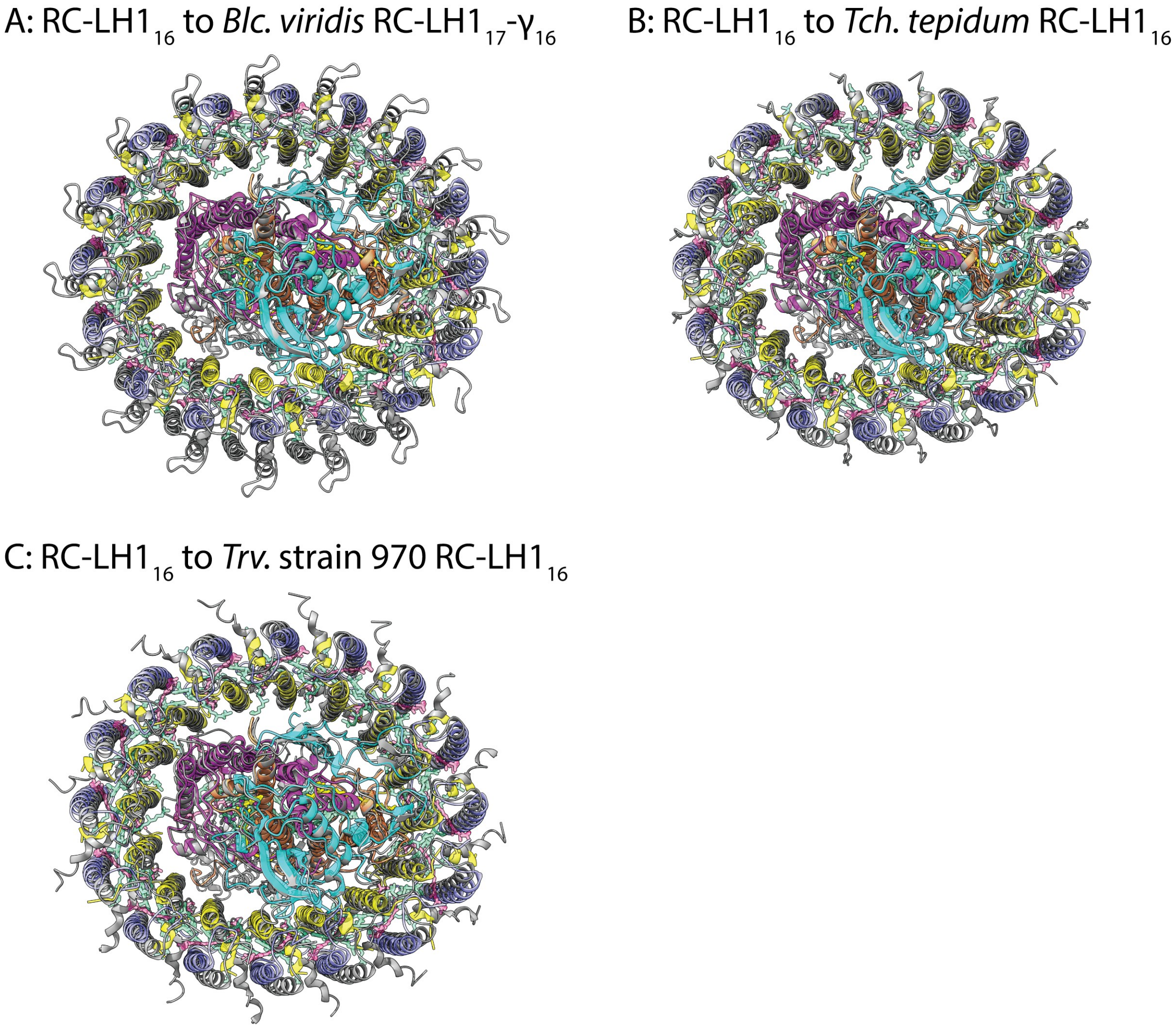

Fig. S6. Overlays of the RC-LH1$_{16}$ structure with other closed RC-LH1 complexes in the PDB (see figure labels for details). The core complexes of *Rps. palustris* are colored as in Fig. 1 with the aligned structure in grey. Alignments were generated by aligning the backbone of the L and M RC subunits in ChimeraX.

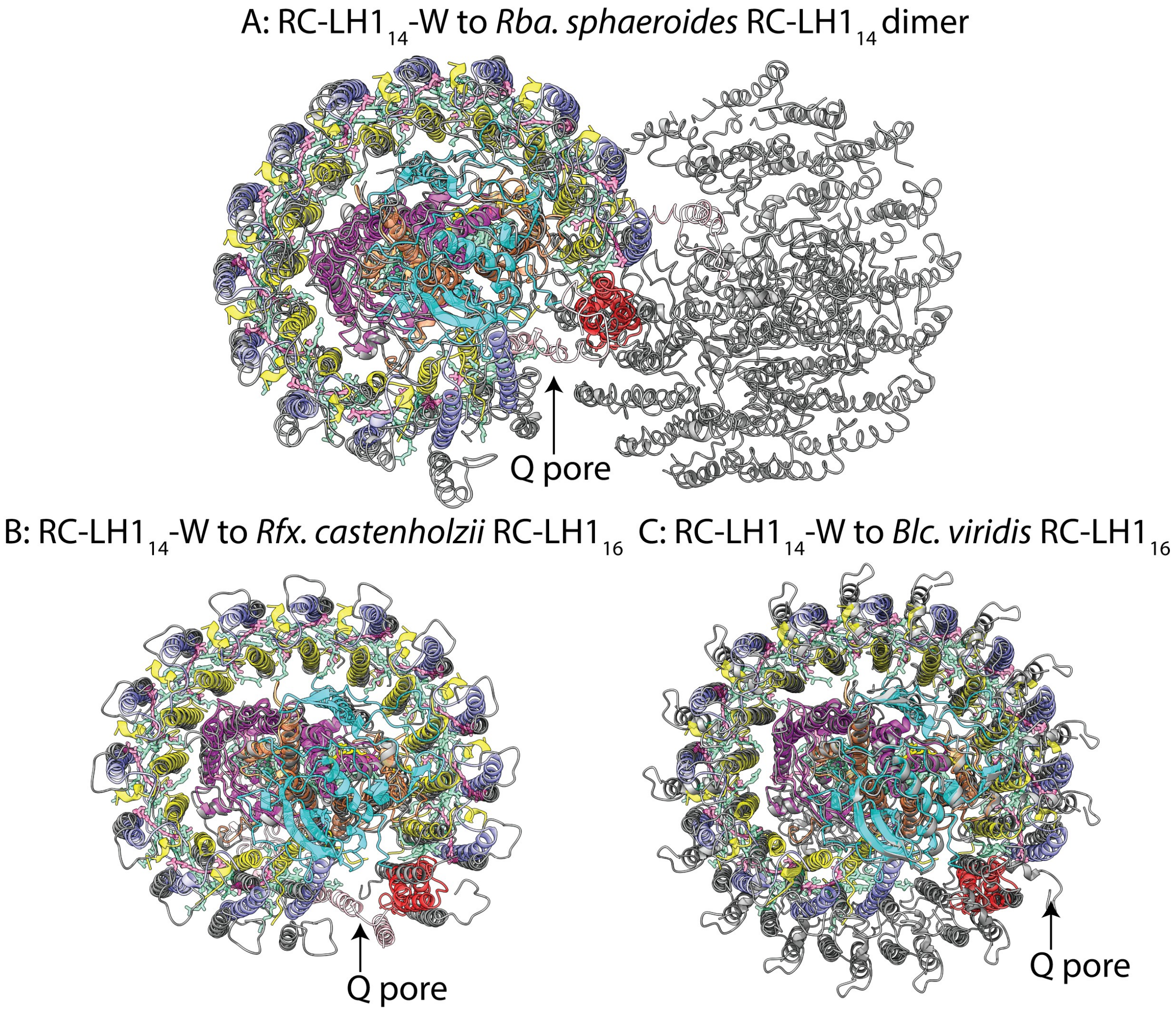

Fig. S7. Overlays of the RC-LH1$_{14}$-W structure with other RC-LH1 complexes in the PDB (see figure labels for details). The core complexes of *Rps. palustris* are colored as in Fig. 1 with the aligned structure in grey except the pore-forming protein PufX in the *Rba. sphaeroides* RC-LH1, and proteins X and C in the *Rfx. castenholzii* RC-LH1, which are shown in pink. Alignments were generated by aligning the backbone of the L and M RC subunits in ChimeraX.

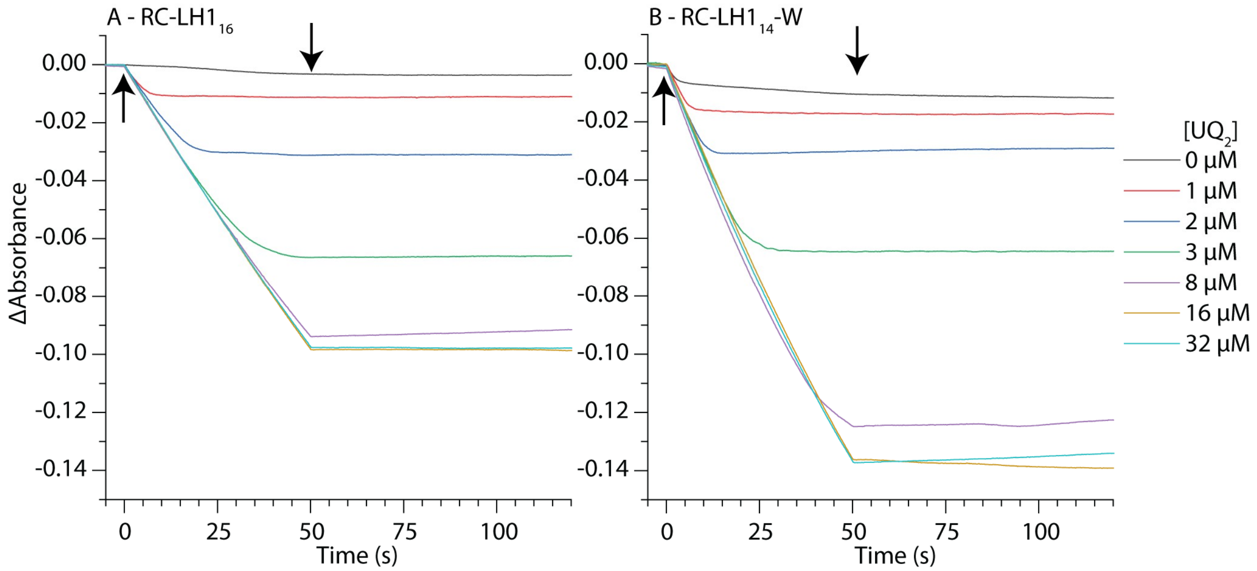

Fig. S8. Absorbance changes at 550 nm upon illumination of RC-LH1 complexes in solution with reduced cytochrome c_2 and UQ_2. Samples were illuminated with a 1 W 880 nm LED driven at 900 mW for 50 s. The assay contained core complexes adjusted to the Q_y maximum absorbance (~880 nm) of 0.11 (corresponding to 30 and 34 nM RC-LH1$_{16}$ and RC-LH1$_{14}$-W, respectively), 30 µM cytochrome c_2 and the indicated concentration of UQ_2. Upward arrows indicate the beginning of the illumination period (0 s) and downward arrows indicate the end of the illumination period (50 s).

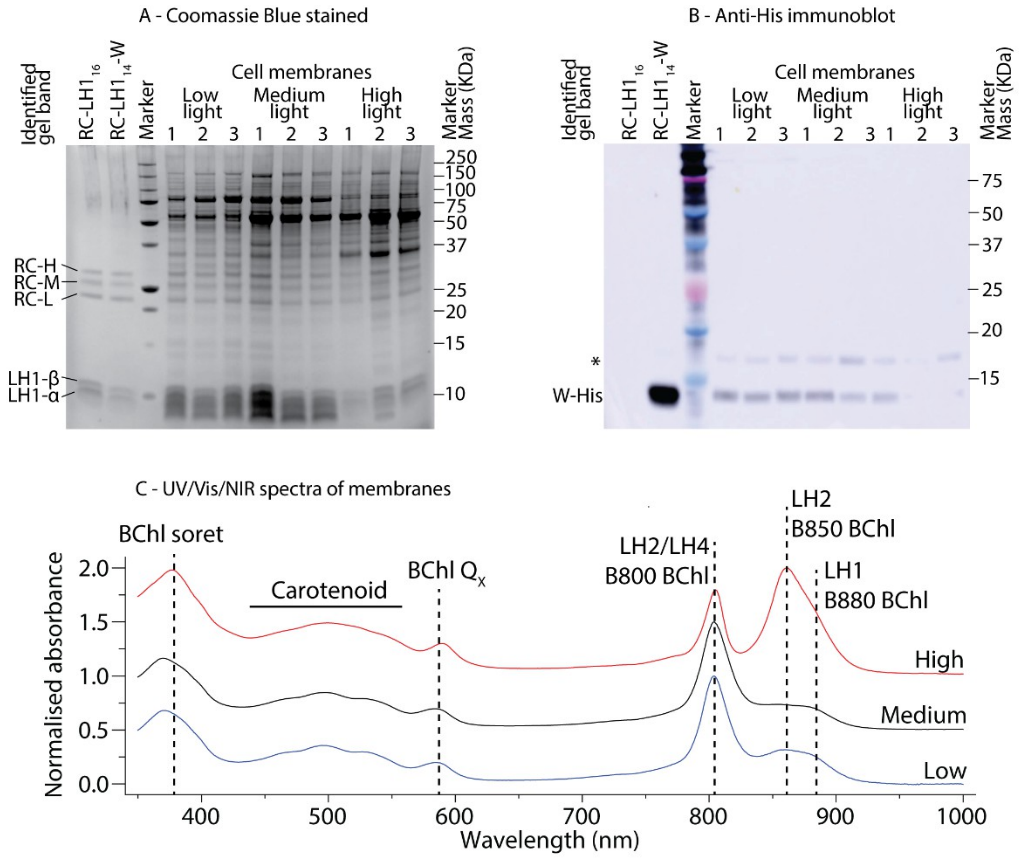

Fig. S9. Quantification of protein-W in RC-LH1 core complexes from cells grown under differing light intensities. Panel A shows a Coomassie-stained SDS-PAGE gel. Panel B shows the corresponding anti-His immunoblot. In both panels, purified RC-LH1$_{16}$ and RC-LH1$_{14}$-W are loaded to the left of the MW marker and three independent membrane preparations from cells grown at low (10 µM m^{-2} s^{-1}), medium (30 µM m^{-2} s^{-1}) and high (300 µM m^{-2} s^{-1}) illumination for 72 h to the right of the MW marker. Bands are labelled with their corresponding proteins and non-specific signals from the anti-His antibody are labelled with an asterisk. The intensities of the RC-L band and the W-His band were integrated and used to calculate RC-L to W-His ratios relative to the pure RC-LH1$_{14}$-W complex, in which they are present in a 1:1 molar ratio. The resulting RC-L to W-His ratios are shown in Fig. 2F. Panel C shows UV/Vis/NIR spectra of one membrane preparation from each light intensity normalized to the maximal absorbance between 750 and 950 nm with absorption bands labeled.

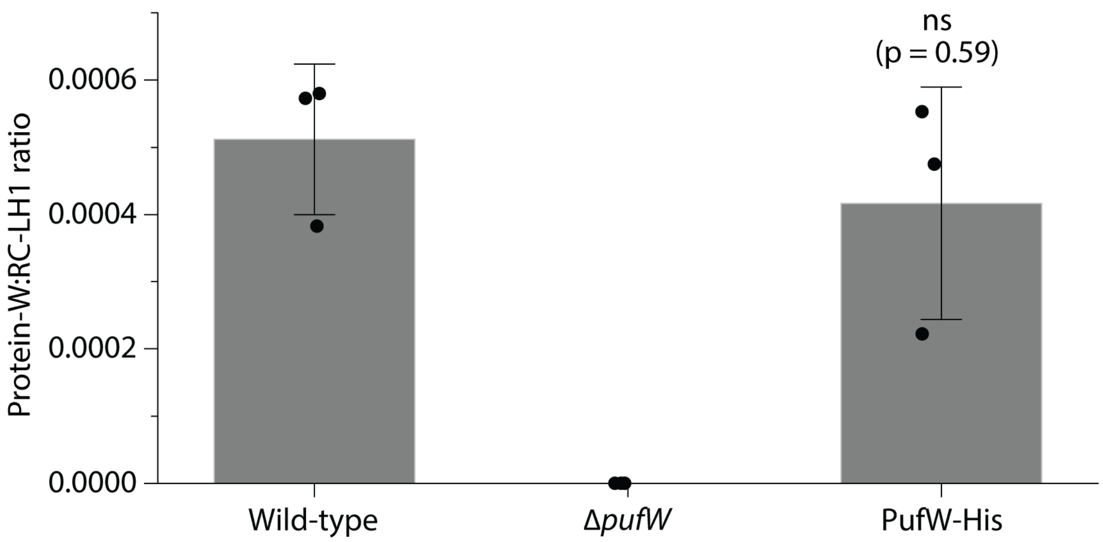

Fig. S10. Quantification of protein-W in RC-LH1 core complexes using mass spectrometry. Wild-type, Δ*pufW* and PufW-His cells were grown in M22 media under 30 μM m^{-2} s^{-1} illumination. The relative abundance of protein-W and the combined RC-LH1 subunits in purified membranes was determined from the intensity values for the individual subunits, extracted from tryptic peptide ion counts by MaxQuant (see Materials and Methods), as shown in Table S3. Error bars represent standard deviation from the mean of three biological replicates from three separate cultures (overlaid in black circles) and the p-value is derived from a paired, 2-tailed Student's t-test vs. the wild-type replicates with 'ns' indicating that the difference is not significant.

Table S1. Change in gene expression for orthologous genes in 17 *R. palustris* strains exposed to low light and high light intensity

Note 1. All strains were grown in nitrogen-fixing medium with 20 mM acetate, Wolfe's vitamins, and 10 μM vanadium chloride

Note 2. Numbers highlighted in red were more highly expressed in cells exposed to high light intensity (ratio of low light intensity/high light intensity ≤0.5)

Note 3. Numbers highlighted in green were more highly expressed in cells exposed to low light intensity (ratio of low light intensity/high light intensity ≥2)

orthoMCL_id	0001L	1a1	CGA009	CGA010	AP1	ATCC17007	BIS3	CEA001	DCP3	DSM126	DSM8283	KD1	NCIB8288	RCH350	RCH500	RSP24	TIE1	Gene name	Protein Name
orthoMCL0863	1.5	1.9	1.4	1.9	1.5	1.2	1.5	1.9	1.7	2.2	1.7	2.0	2.0	2.1	1.8	1.5	2.4	RPA1548	H subunit of photosynthetic reaction center complex
orthoMCL0845	1.3	1.7	1.0	1.5	1.6	0.8	1.1	2.0	1.8	1.9	1.4	1.8	1.9	1.9	1.8	1.4	1.8	RPA1527	photosynthetic reaction center L subunit
orthoMCL0846	1.3	1.7	1.2	1.9	1.6	1.0	1.2	2.0	1.7	2.1	1.4	1.8	2.0	2.0	2.0	1.4	2.0	RPA1528	photosynthetic reaction center M protein
orthoMCL0843	1.4	1.9	1.5	1.9	1.5	1.5	2.0	2.2	1.7	2.1	2.0	2.1	2.1	2.0	1.9	1.4	1.9	RPA1525	light-harvesting complex 1 beta chain
orthoMCL0844	1.4	2.0	1.5	2.3	1.6	1.5	2.1	1.8	1.7	2.0	2.1	2.1	2.1	1.9	1.8	1.3	1.6	RPA1526	light-harvesting complex 1 alpha chain
orthoMCL1394	0.2	0.1	0.1	0.1	0.2	0.2	0.2	0.2	0.1	0.2	0.2	0.1	0.2	0.2	0.1	0.1	0.1	RPA4402	hypothetical protein

Fig. S11. Transcriptomic data for mRNAs encoding the RC and LH1 subunits and protein-W (RPA4402). Green shows a >2-fold increase in expression under low light (i.e. more highly expressed under low light) and red indicates a >2-fold reduction in expression under low light (i.e. more highly expressed under high light). Adapted from Fixen *et al.* 2019 (*23*). Data are shown for 17 *Rps. palustris* strains, including CGA009 utilized in this study.

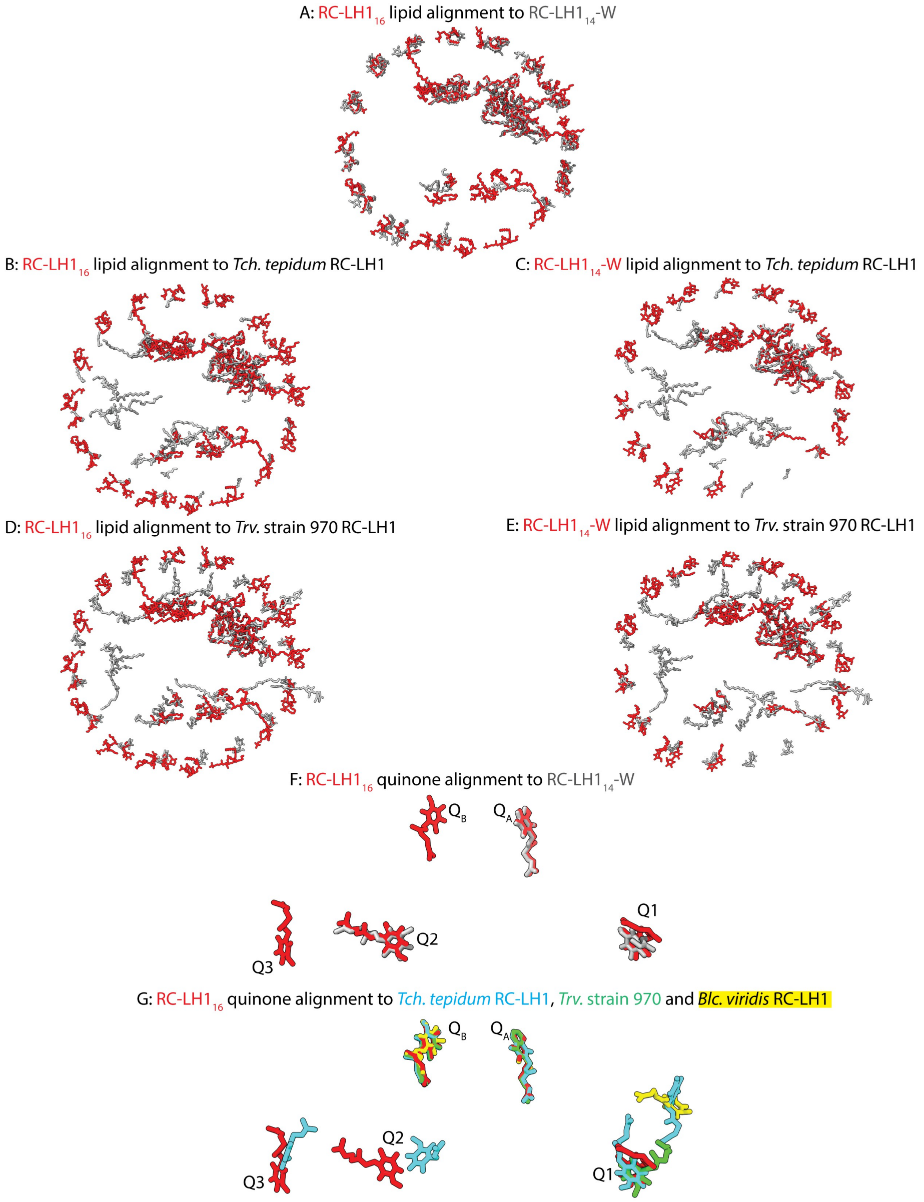

Fig. S12. Structural alignments of resolved lipids, detergents and quinones. Panel A shows alignment of lipids and detergents resolved in the RC-LH1$_{16}$ structure (red) with those from RC-LH1$_{14}$-W (grey). Panels B and C show alignment of lipids and detergents from the RC-LH1$_{16}$ (B) and RC-LH1$_{14}$-W (C) structures with those resolved in the *Tch. tepidum* RC-LH1 structure (PDB 5Y5S) (*9*). Lipids and detergents in the *Rps. palustris* structures are shown in red and the *Tch. tepidum* lipids and detergents are in grey. Panels D and E show alignment of lipids and detergents from the RC-LH1$_{16}$ (D) and RC-LH1$_{14}$-W (E) structures (red) with those resolved in the *Trv.* strain 970 RC-LH1 structure (grey) (PDB 7C9R) (*12*). Panel F shows resolved quinones from RC-LH1$_{16}$ (red) overlaid with those from RC-LH1$_{14}$-W (grey). Panel G shows overlays of resolved quinones from the RC-LH1$_{16}$ in red, *Tch. tepium* RC-LH1 in blue (PDB 5Y5S) (*9*) , *Trv.* strain 970 in green (PDB 7C9R) (*12*) and *Blastochloris* (*Blc.*) *viridis* in yellow (PDB 6ET5) (*10*). Quinones from the RC-LH1$_{16}$ structure are labelled as in Fig. 5. For clarity, the tails of the quinone groups have been truncated to two isoprene units. All alignments were generated by aligning the RC L and M subunits in ChimeraX.

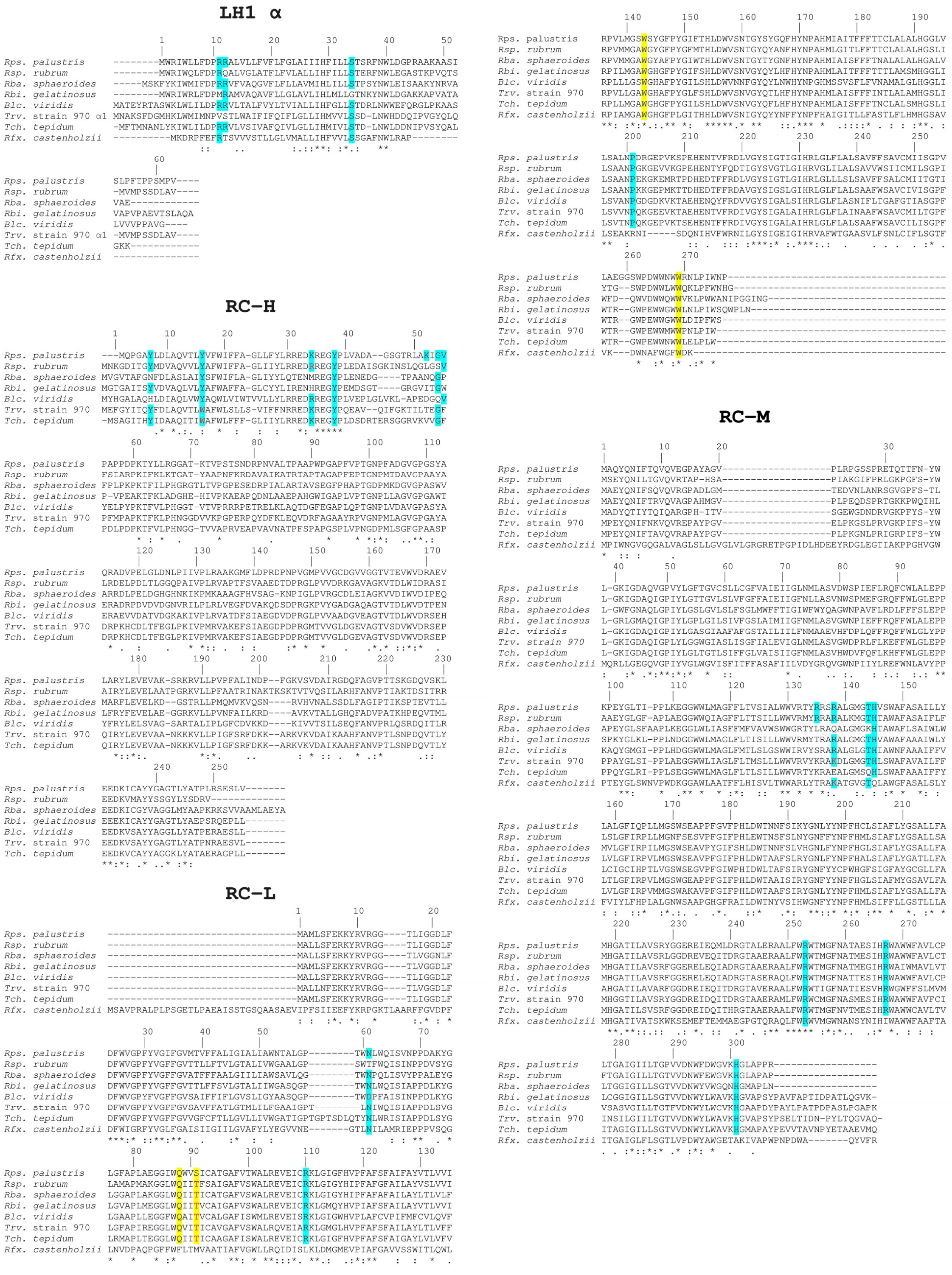

Fig. S13. Alignments of RC-LH1 peptides from *Rps. palustris*, *Rhodospirillum* (*Rsp.*) *rubrum*, *Rba. sphaeroides*, *Rubrivivax* (*Rvi.*) *gelatinosus*, *Blc. viridis*, *Trv.* strain 970, *Tch. tepidum* and *Rfx. castenholzii*. Conserved residues which hydrogen-bond to lipid head-groups in the RC-$LH1_{16}$ structure are highlighted in cyan, with conservative substitutions in red text. Yellow highlights show conserved residues for the binding of sequestered quinone molecules.

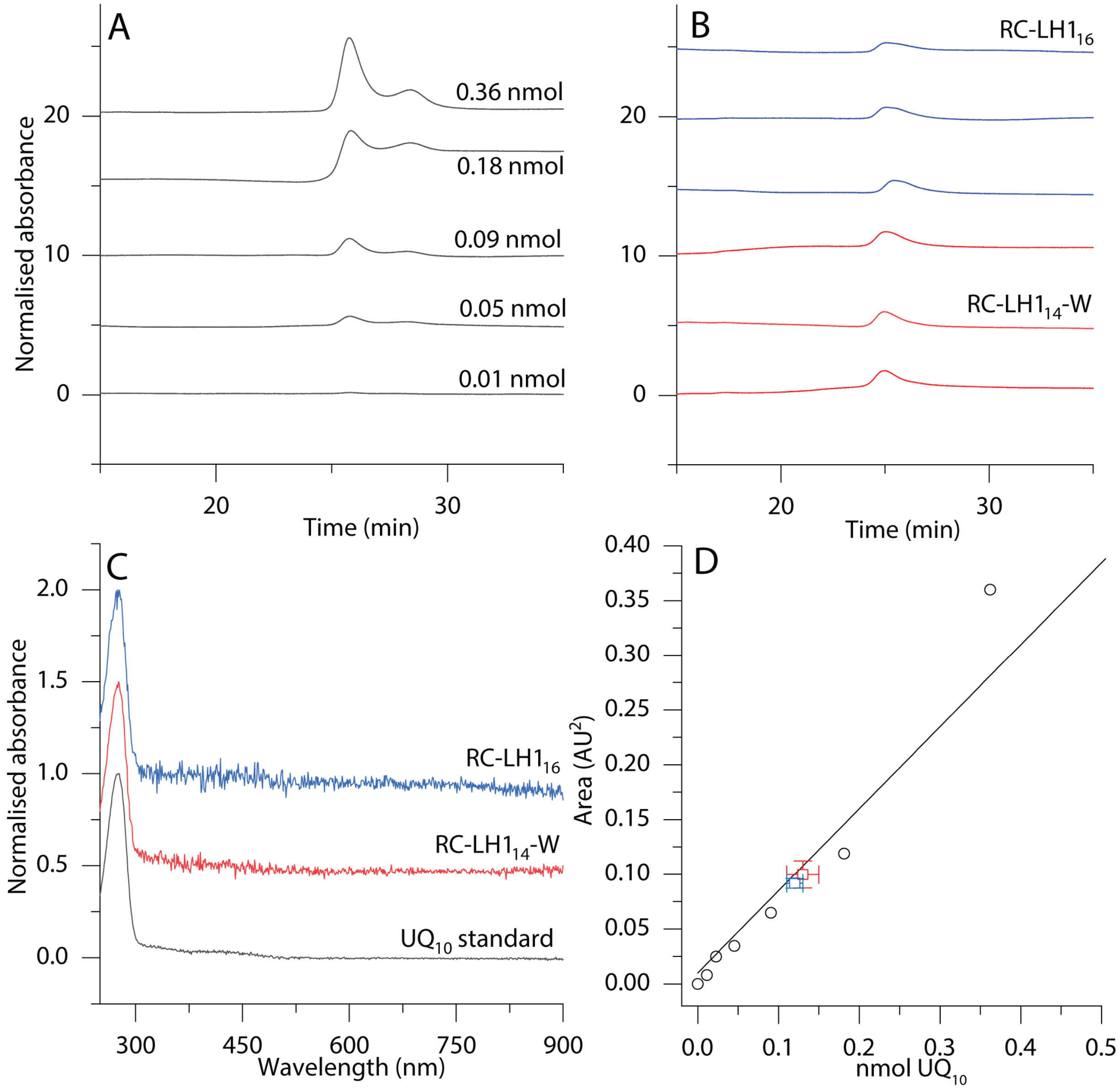

Fig. S14. RP-HPLC quantification of UQ_{10}. Panel A shows selected chromatograms of pure UQ_{10} standards at the indicated concentrations monitoring absorbance at 275 nm. The double peak at 25.5 and 28 minutes corresponds to UQ_{10}. Panel B shows chromatograms of three RC-LH1$_{14}$-W (red) and three RC-LH1$_{16}$ samples (blue) loaded in quantities of 0.023 and 0.020 nmol, respectively. Panel C shows normalized spectra of the 25.5 min peak for the 0.72 nmol standard and one run for each set of the RC-LH1 samples. Panel D shows the calibration curves generated from integrated areas of the UQ_{10} standards, from which the amount of UQ_{10} in the RC-LH1 complexes was calculated. Calculated values for the samples are shown with red (RC-LH1$_{14}$-W) and blue (RC-LH1$_{16}$) squares.

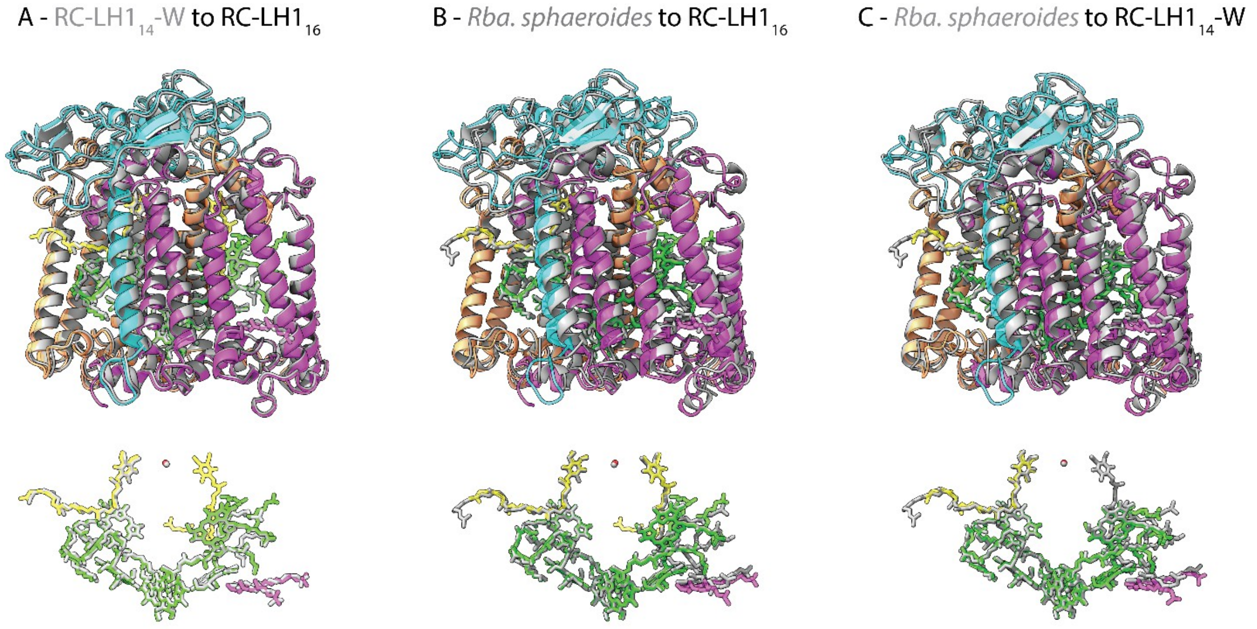

Fig. S15. Alignments of RC structures. Panel A shows alignment of the RC-LH1$_{14}$-W RC (colored grey) to the RC of the RC-LH1$_{16}$ complex (in color). Panel B shows an alignment of the RC from *Rba. sphaeroides* (PDB ID: 3I4D) (grey) to the RC of the RC-LH1$_{16}$ complex (color). Panel C shows alignment of the RC from *Rba. sphaeroides* (PDB ID: 3I4D) (grey) to the RC of the RC-LH1$_{14}$-W complex (color). Top panels show the protein in cartoon representation with cofactors as sticks, and bottom panels show the same view with the protein removed. Alignments of the peptide backbones of the L, H and M chains were performed in ChimeraX. In the colored structures, chain L is in orange, M is in magenta and H is in cyan, BChls and BPhs are green, carotenoids are purple, UQ$_{10}$ molecules are yellow and non-heme iron is red. RMSD values were 0.539 Å for the alignment in panel A, 0.877 Å for panel B and 0.633 Å for panel C.

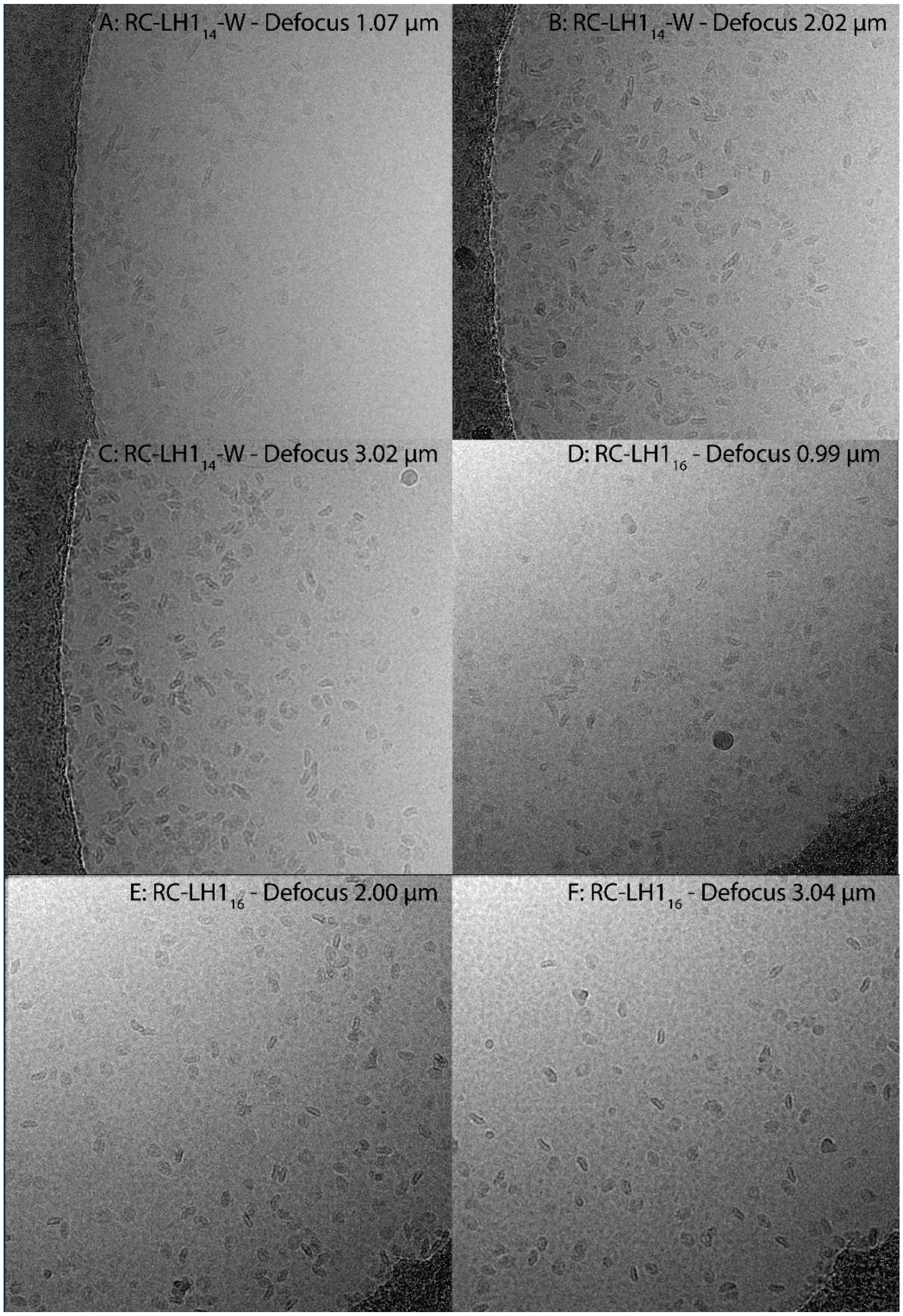

Fig. S16. Typical motion corrected cryo-TEM micrographs. Selected micrographs are shown for RC-LH1$_{14}$-W (Panels A-C) and RC-LH1$_{16}$ (Panels D-F). Micrographs are selected at defocus values of approximately 1, 2 and 3 µm to show the full defocus range over which images were collected.

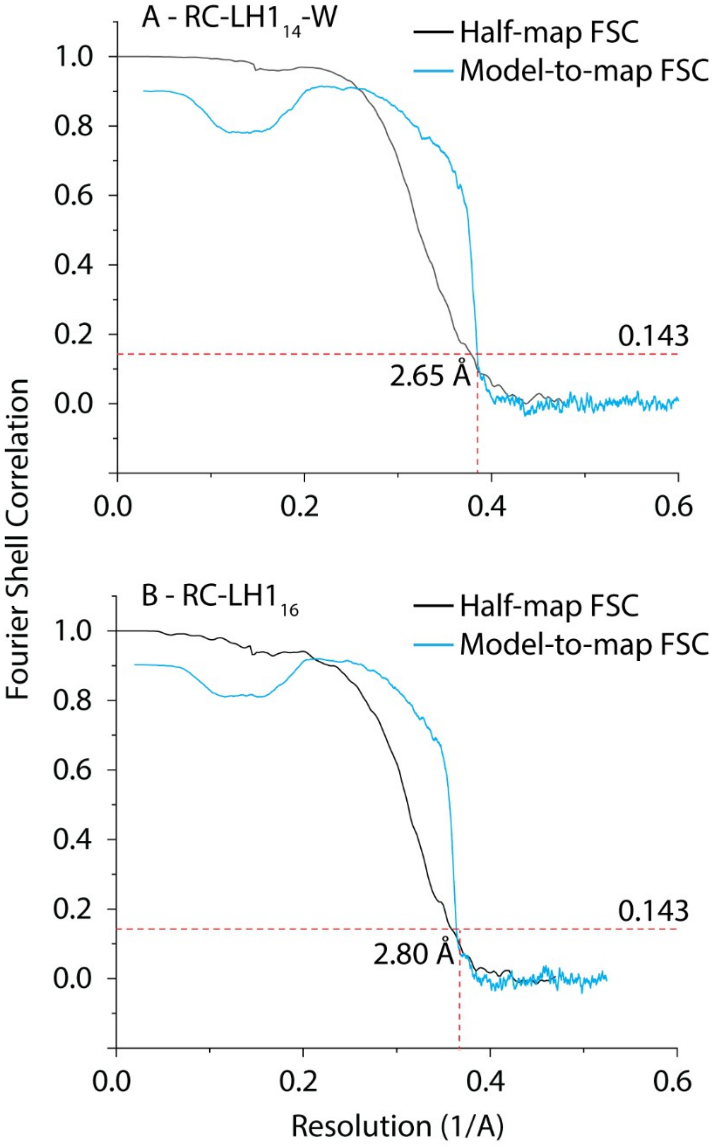

Fig. S17. Resolution determination using half-map and model-to-map fourier shell correlation (FSC) curves with a 0.143 threshold. Half-map (black) and model-to-map (blue) FSC curves for RC-LH1$_{14}$-W (panel A) and RC-LH1$_{16}$ (panel B). The resolution, as indicated on the graph, was determined at a threshold of 0.143 (red dashed line) from the half-map FSCs. Model-to-map FSCs reach this threshold at similar values, suggesting good agreement between the map and model.

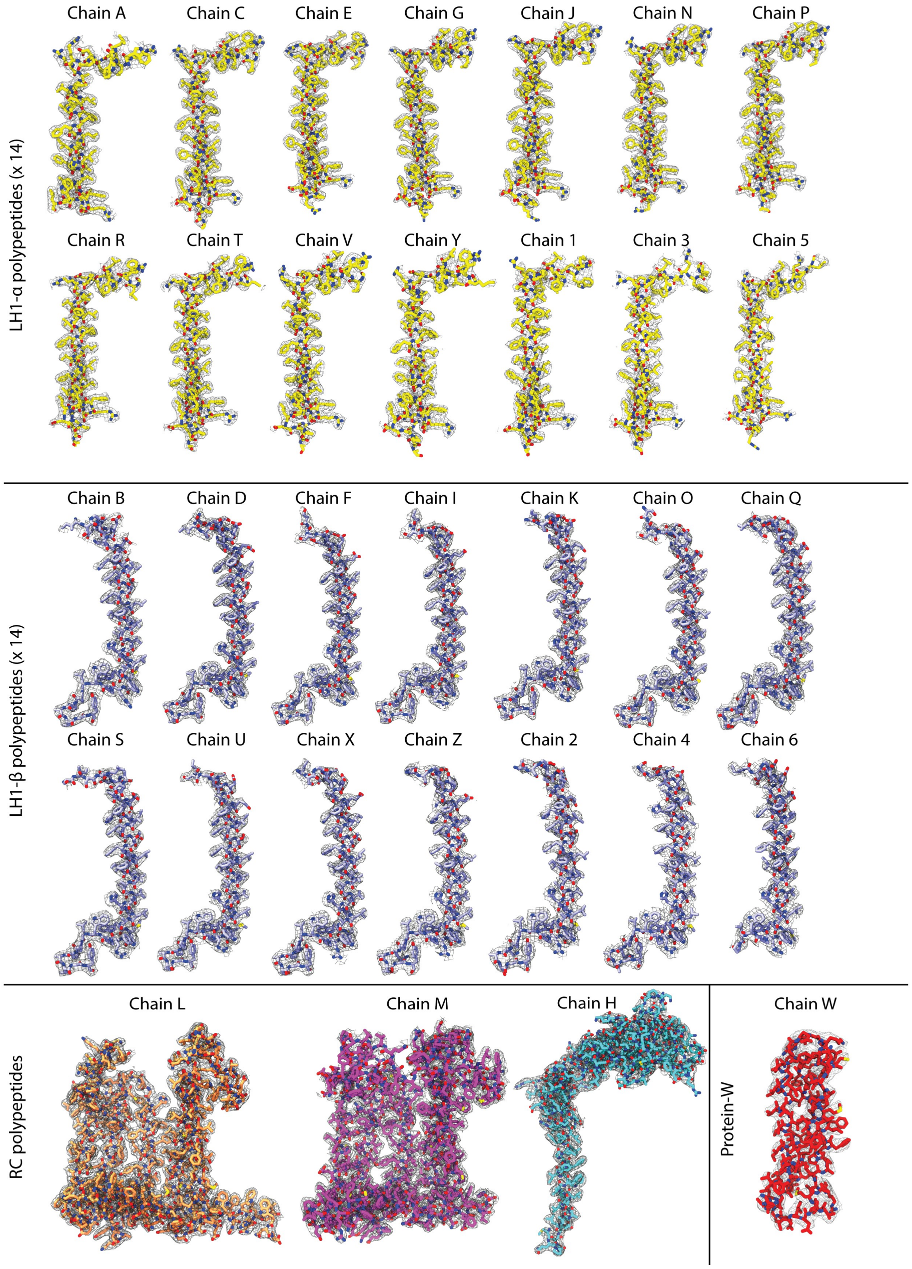

Fig. S18. Models for the polypeptides and their corresponding density from the RC-LH1$_{14}$-W complex. Sections are labelled to indicate which subunit each polypeptide is associated with and each polypeptide is labelled with the residue name, chain ID and residue number. Figure produced using ChimeraX.

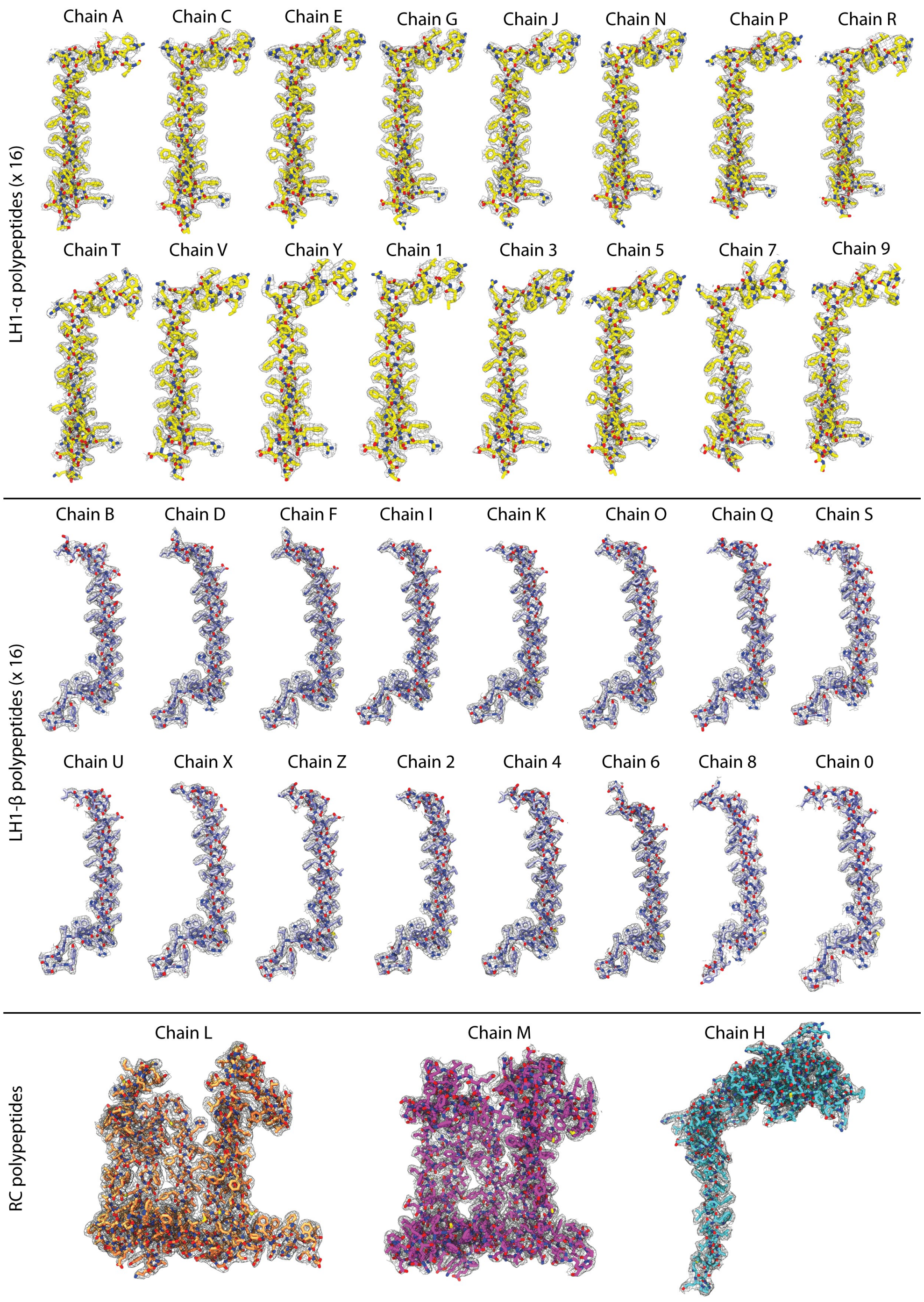

Fig. S19. Models for the polypeptides and their corresponding density from the RC-LH1$_{16}$ complex. Sections are labelled to indicate which subunit each polypeptide is associated with and each polypeptide is labelled with the residue name, chain ID and residue number. Figure produced using ChimeraX.

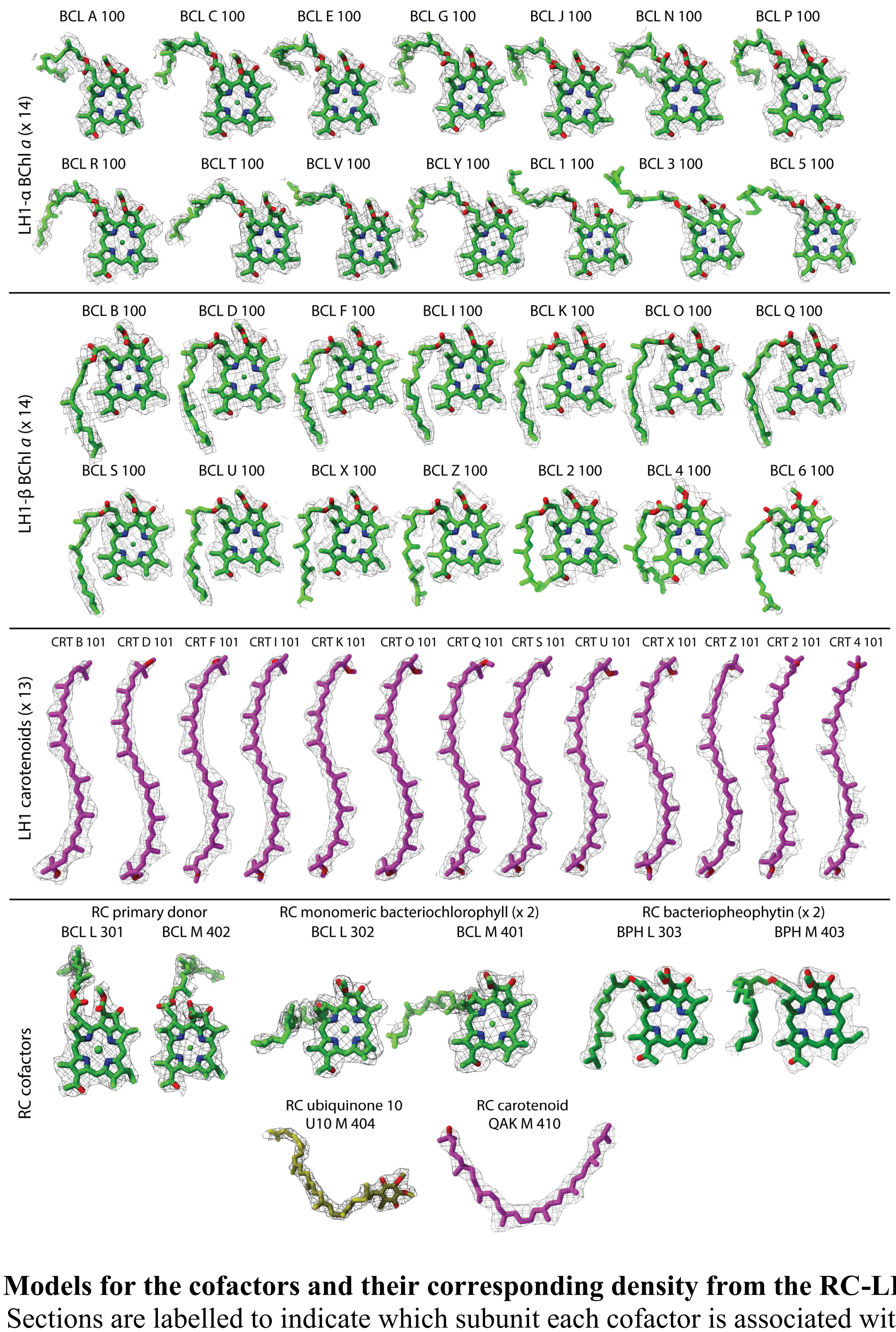

Fig. S20. Models for the cofactors and their corresponding density from the RC-LH1$_{14}$-W complex. Sections are labelled to indicate which subunit each cofactor is associated with and each cofactor is labelled with the residue name, chain ID and residue number. Figure produced using ChimeraX. BCL = BChl, CRT = spirilloxanthin, QAK = *cis*-3,4-dehydrorhodopin, U10 = UQ$_{10}$.

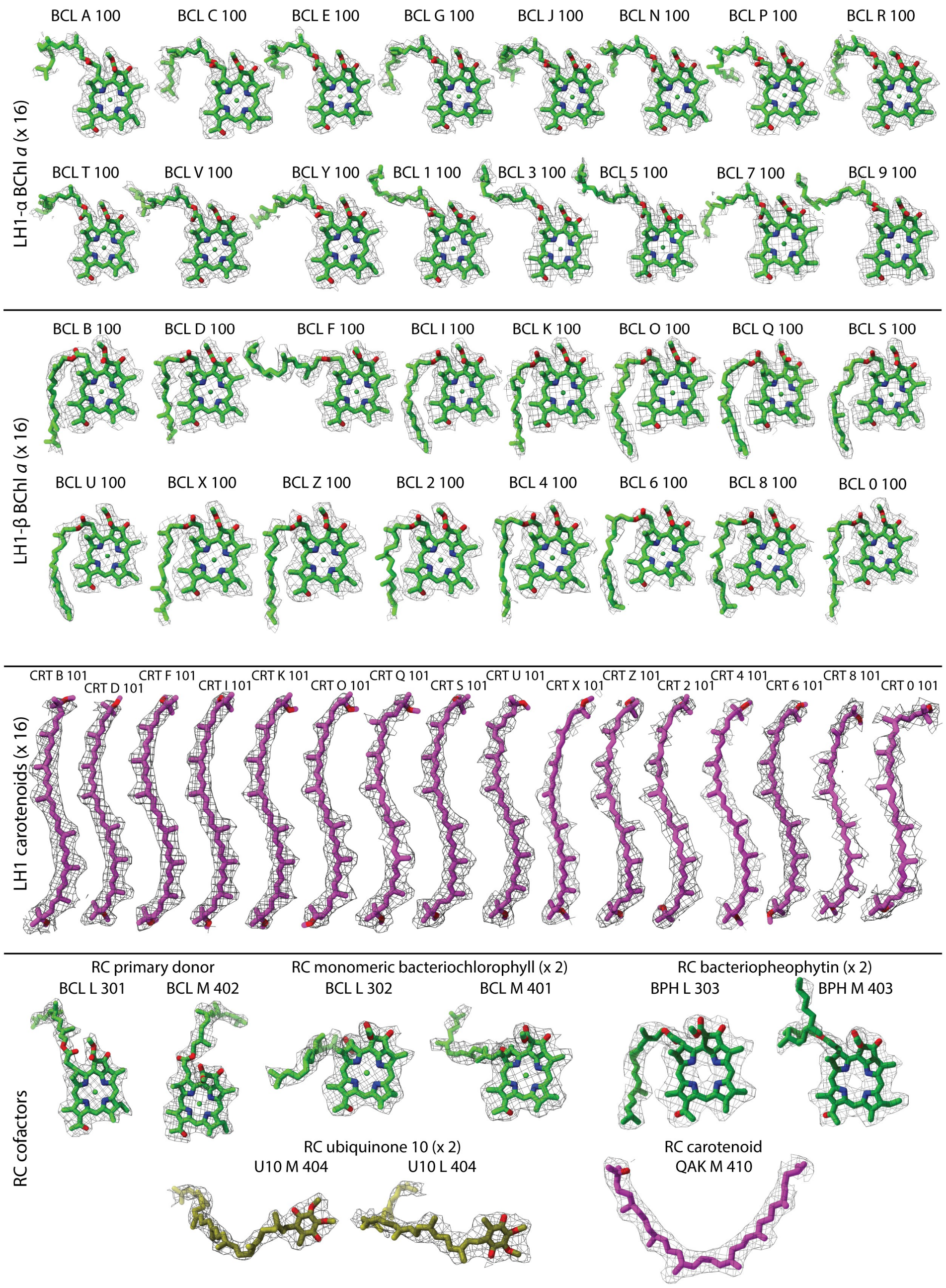

Fig. S21. Models for the cofactors and their corresponding density from the RC-LH1$_{16}$ complex. Sections are labelled to indicate which subunit each cofactor is associated with and each cofactor is labelled with the residue name, chain ID and residue number. Figure produced using ChimeraX. BCL = BChl, CRT = spirilloxanthin, QAK = *cis*-3,4-dehydrorhodopin, U10 = UQ_{10}.

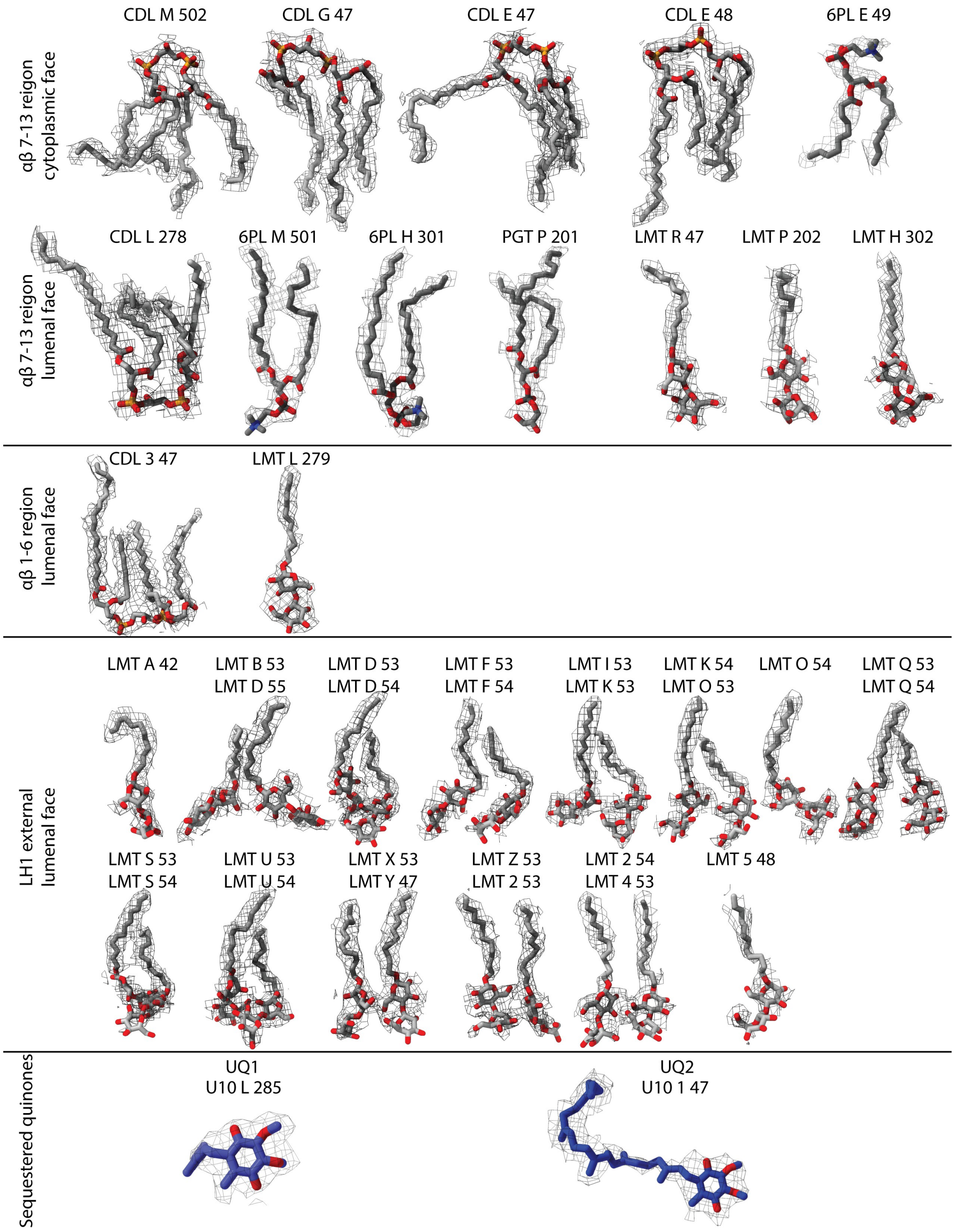

Fig. S22. Models for the lipids, detergents and sequestered quinones and their corresponding density from the RC-LH1$_{14}$-W complex. Sections are labelled to indicate the area in which each molecule is located and each molecule is labelled with the residue name, chain ID and residue number. Figure produced using ChimeraX. CDL = CDL, 6PL = POPC, PGT = POPG, LMT = βDDM, U10 = UQ_{10}.

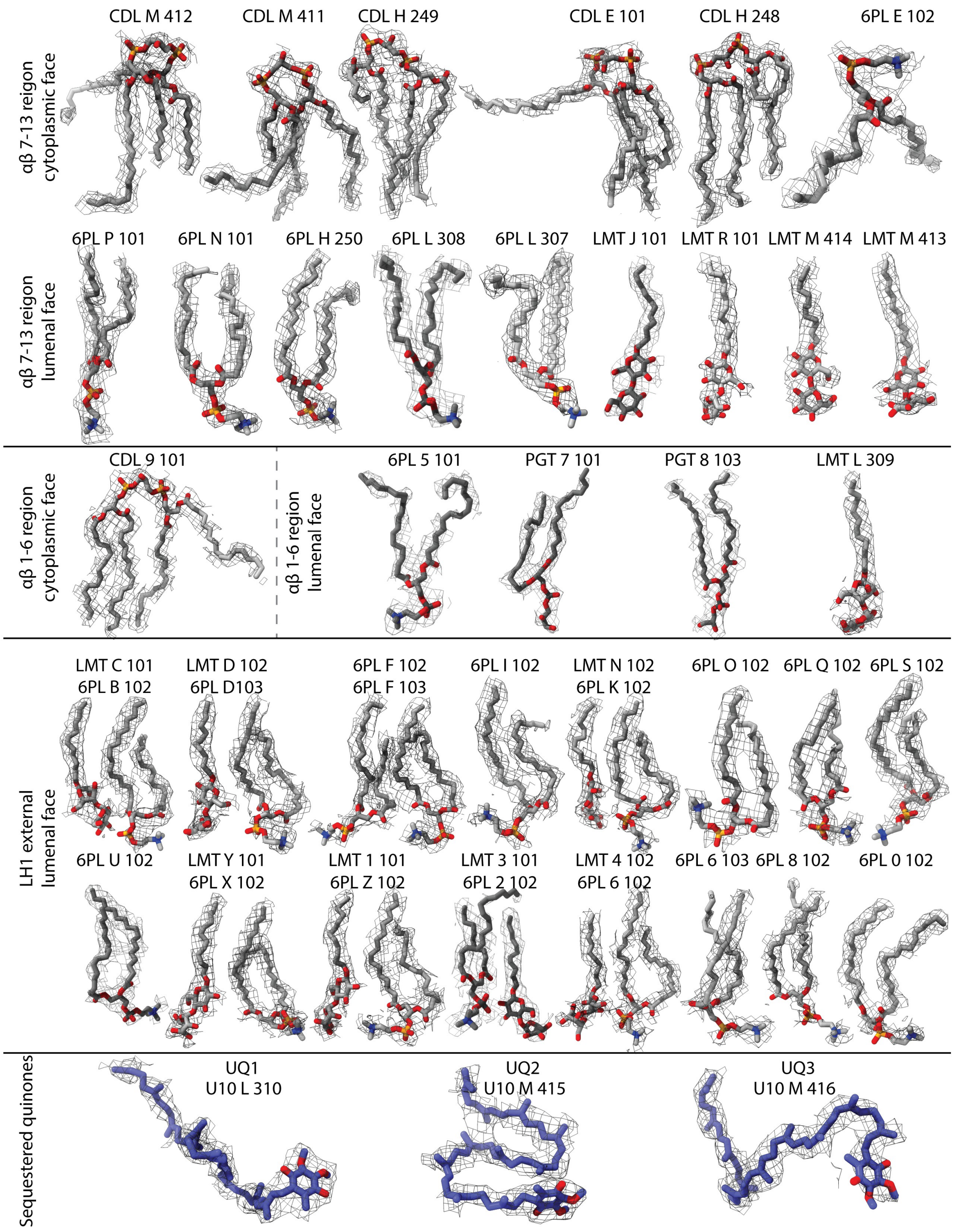

Fig. S23. Models for the lipids, detergents and sequestered quinones and their corresponding density from the RC-LH1$_{16}$ complex. Sections are labelled to indicate the area in which each molecule is located and each molecule is labelled with the residue name, chain ID and residue number. Figure produced using ChimeraX. CDL = CDL, 6PL = POPC, PGT = POPG, LMT = βDDM, U10 = UQ$_{10}$.

Table S1. Cryo-EM data collection, refinement and validation statistics

	RC-LH1$_{14}$-W (EMD-11081) (PDB-6Z5S)	RC-LH1$_{16}$ (EMD-11080) (PDB-6Z5R)
**Data collection and processing**		
Magnification	130,000 x	130,000 x
Electron exposure (e^-/Å^2)	46.2	55.2
Defocus range	-1 to -3 µm	-1 to -3 µm
Pixel size (Å)	1.048	1.065
Symmetry imposed	C1	C1
Initial particle images (no.)	849,359	476,547
Final particle images (no.)	377,703 (44.5%)	260,752 (54.7%)
Map resolution (Å)	2.65	2.80
FSC threshold	0.143	0.143
**Refinement**		
Initial model used	RELION *de novo* model	RELION *de novo* model
Model resolution (Å)	2.60	2.80
FSC threshold	0.143	0.143
Model resolution range (Å)	~2.5-5.5	~2.5-6.0
Map sharpening *B* factor (Å^2)	-38.73*	-47.31*
Model Composition		
Non-hydrogen atoms	22795	25048
Protein residues	2214	2336
Ligands	91	105
B factors (Å^2)		
Protein	25.72 – 127.22 (54.21)	27.47 – 104.18 (48.31)
Ligand	31.09 – 85.08 (49.41)	30.91 – 74.19 (48.84)
R.M.S deviations (PHENIX)		
Bond lengths (Å)	0.009 (0)	0.006 (0)
Bond angles (°)	2.784 (470)	2.780 (550)
Validation		
MolProbity score	2.05	1.79
Clashscore	14.49	10.74
Rotamer outliers (%)	0.00	0.00
Ramachandran plot		
Favored (%)	94.34	96.36
Allowed (%)	5.66	3.64
Outliers (%)	0.00	0.00

*Values for initial maps from RELION used for initial model building and refinement. Final maps were locally sharpened using LocScale (*50*).

Table S2. Fitted rates and amplitudes for transient absorption of RC-LH1$_{14}$-W and RC-LH1$_{16}$ at 895 nm and 591 nm (presented in Fig. S4) and rates from global fitting (presented in Fig. S5).

		Amp 1 (AU)	τ_1 (ps)	Amp 2 (AU)	τ_2 (ps)	Amp 3 (AU)	τ_3 (ps)	Amp4 (AU)	τ_4 (ps)
RC-LH1$_{14}$-W	895 nm	-0.003	2.9	-0.019	43	-0.004	708	-0.002	5161
	591 nm	-0.0001	1.1	-0.0002	35	-0.0001	317	-0.0001	7308
	Global	-	2.5	-	42	-	605	-	6000
				Avg	***40±4***				
RC-LH1$_{16}$	895 nm	-0.006	2.8	-0.030	47	-0.002	1293	-0.001	11334
	591 nm	-	-	-0.0003	41	-	-	-0.00004	2339
	Global	-	2.4	-	44	-	499	-	6200
	Avg			***Avg***	***44±3***				

τ_1 = LH1 excited state (LH1*) singlet-singlet annihilation

τ_2 = LH1 to RC energy transfer

τ_3 = LH1* deactivation to ground state and/or RC $P^+H_A^- \rightarrow P^+Q_A^-$ ET

τ_4 = RC $P^+H_A^-$ charge recombination

Table. S3.

Mass spectral ion intensities for the RC-H, -M and -L subunits, LH 1-α and -β subunits and protein-W in wild-type, Δ*pufW* and PufW-His membranes. Proteins extracted from membranes isolated from the three strains (n = 3 biological replicates from three separate cultures) of *Rps. palustris* were digested with a combination of endoproteinase Lys-C and trypsin. The resultant peptides (500 ng) were analyzed by nano-flow reverse phase chromatography coupled to mass spectrometry. (A): Mass spectral ion intensities for peptides mapping to the RC-LH1-W subunits were extracted by MaxQuant. Only peptides validated by product ion spectra in all nine analyses were utilized. Intensities for peptides containing Met are shown as the sum of both native and sulfoxide forms. (B): Summed peptide ion intensities were used to generate Fig. S13.The mass spectral data-files and MaxQuant results are available from the ProteomeXchange Consortium via the PRIDE partner repository (http://proteomecentral.proteomexchange.org) with the data set identifier PXD020402.

(A)

Peptide	Wild-type			Δ*pufW*			PufW-His		
	1	2	3	1	2	3	1	2	3
**RC-H**									
ADV-PLR	4.29E+09	4.04E+09	4.10E+09	3.24E+09	3.44E+09	3.56E+09	3.48E+09	3.90E+09	3.51E+09
DPN-VDR	4.30E+08	4.67E+08	4.96E+08	2.13E+08	2.55E+08	2.29E+08	2.19E+08	2.24E+08	2.48E+08
DPN-LAR	1.98E+08	3.94E+08	3.94E+08	3.16E+08	5.36E+08	3.06E+08	2.24E+08	3.14E+08	1.09E+08
EGY-GTR	6.70E+07	7.16E+07	7.72E+07	3.54E+07	3.50E+07	3.16E+07	4.13E+07	3.82E+07	4.03E+07
GDQ-TSK	5.21E+08	5.18E+08	5.49E+08	3.44E+08	3.48E+08	3.59E+08	3.39E+08	3.18E+08	3.21E+08
GMF-DPR	2.81E+08	3.06E+08	3.19E+08	1.56E+08	1.62E+08	1.65E+08	1.58E+08	1.44E+08	1.55E+08
ICA-PLR	4.62E+08	4.54E+08	4.24E+08	8.78E+08	8.46E+08	9.05E+08	8.89E+08	9.36E+08	9.69E+08
ICA-SLV	3.60E+07	3.42E+07	4.07E+07	2.44E+08	2.64E+08	2.57E+08	1.81E+08	1.83E+08	2.03E+08
IGV-DPK	4.63E+08	4.52E+08	4.49E+08	3.30E+08	3.32E+08	3.33E+08	2.86E+08	2.87E+08	3.08E+08
KRV-FGK	3.56E+07	3.00E+07	2.82E+07	1.69E+08	2.01E+08	1.99E+08	1.68E+08	1.54E+08	1.62E+08
LEE-PLR	4.19E+06	4.17E+06	3.15E+06	9.87E+06	9.69E+06	1.10E+07	1.89E+07	1.95E+07	1.88E+07
REG-GTR	2.07E+08	2.00E+08	2.07E+08	1.35E+08	1.45E+08	1.48E+08	1.36E+08	1.35E+08	1.44E+08
RVL-FGK	7.41E+08	5.98E+08	6.59E+08	1.20E+09	1.30E+09	1.22E+09	1.17E+09	1.26E+09	1.21E+09
TVP-AQR	1.85E+09	1.47E+09	1.85E+09	1.30E+09	1.29E+09	1.22E+09	1.18E+09	1.19E+09	1.21E+09
VLL-FGK	6.79E+08	6.43E+08	6.00E+08	8.05E+08	8.47E+08	9.08E+08	9.51E+08	1.01E+09	9.43E+08
VSV-AIR	4.72E+08	4.71E+08	5.17E+08	3.03E+08	3.24E+08	3.13E+08	3.10E+08	3.29E+08	3.20E+08
VSV-TSK	3.23E+08	3.32E+08	3.81E+08	4.39E+08	4.78E+08	4.82E+08	3.38E+08	3.43E+08	3.10E+08
YLE-VAK	7.11E+08	8.21E+08	9.12E+08	7.11E+08	6.46E+08	7.11E+08	6.19E+08	6.59E+08	6.31E+08
**RC-M**									
AQY-SPR	2.75E+09	2.82E+09	2.87E+09	1.98E+09	2.11E+09	2.08E+09	2.10E+09	2.25E+09	2.11E+09
EIE-LDR	1.87E+08	1.95E+08	2.09E+08	8.18E+07	8.02E+07	8.27E+07	9.47E+07	9.69E+07	9.96E+07
ETQ-LGK	1.26E+08	1.28E+08	1.51E+08	9.54E+07	9.73E+07	9.26E+07	8.60E+07	9.37E+07	8.35E+07
**RC-L**									
AMLSFEK	3.25E+08	3.23E+08	3.74E+08	2.49E+08	2.40E+08	2.56E+08	2.40E+08	2.32E+08	2.33E+08
**LH1-α**									
FNW-GPR	2.22E+09	2.12E+09	2.34E+09	1.74E+09	1.67E+09	1.79E+09	1.57E+09	1.57E+09	1.48E+09
**LH1-β**									
SDG-EAK	2.80E+07	2.79E+07	2.90E+07	9.64E+06	1.10E+07	1.04E+07	2.06E+07	2.21E+07	1.95E+07
**Protein-W**									
AGI-LMR	6.67E+06	9.80E+06	1.03E+07	0.00E+00	0.00E+00	0.00E+00	8.19E+06	7.46E+06	3.30E+06

(B)

Subunit	Wild-type			Δ*pufW*			PufW-His		
	1	2	3	1	2	3	1	2	3
RC-H	1.18E+10	1.13E+10	1.20E+10	1.08E+10	1.15E+10	1.14E+10	1.07E+10	1.14E+10	1.08E+10
RC-M	3.07E+09	3.14E+09	3.23E+09	2.15E+09	2.29E+09	2.25E+09	2.28E+09	2.44E+09	2.30E+09
RC-L	3.25E+08	3.23E+08	3.74E+08	2.49E+08	2.40E+08	2.56E+08	2.40E+08	2.32E+08	2.33E+08
LH1-α	2.22E+09	2.12E+09	2.34E+09	1.74E+09	1.67E+09	1.79E+09	1.57E+09	1.57E+09	1.48E+09
LH1-β	2.80E+07	2.79E+07	2.90E+07	9.64E+06	1.10E+07	1.04E+07	2.06E+07	2.21E+07	1.95E+07
Sum (RC-LH1)	1.74E+10	1.69E+10	1.80E+10	1.50E+10	1.57E+10	1.57E+10	1.48E+10	1.57E+10	1.48E+10
Protein-W	6.67E+06	9.80E+06	1.03E+07	0.00E+00	0.00E+00	0.00E+00	8.19E+06	7.46E+06	3.30E+06
W:RC-LH1 ratio	3.83E-04	5.80E-04	5.73E-04	0.00E+00	0.00E+00	0.00E+00	5.53E-04	4.75E-04	2.22E-04
Ratio mean		5.12E-04			0			4.17E-04	
Ratio SD		1.12E-04						1.73E-04	

REFERENCES AND NOTES

1. S. Romagnoli, R. F. Tabita, Carbon dioxide metabolism and its regulation in nonsulfur purple photosynthetic bacteria, in *The Purple Phototrophic Bacteria*, C. N. Hunter, F. Daldal, M. C. Thurnauer, J. T. Beatty, Eds. (Springer Netherlands, 2009), pp. 563–576.

2. B. Masepohl, R. G. Kranz, Regulation of nitrogen fixation, in *The Purple Phototrophic Bacteria*, C. N. Hunter, F. Daldal, M. C. Thurnauer, J. T. Beatty, Eds. (Springer, 2009), pp. 759–775.

3. C. S. Harwood, J. Gibson, Anaerobic and aerobic metabolism of diverse aromatic compounds by the photosynthetic bacterium *Rhodopseudomonas palustris*. *Appl. Environ. Microbiol.* **54**, 712–717 (1988).

4. M. Sener, J. Strumpfer, A. Singharoy, C. N. Hunter, K. Schulten, Overall energy conversion efficiency of a photosynthetic vesicle. *eLife* **5**, e09541 (2016).

5. P. D. Dahlberg, P.-C. Ting, S. C. Massey, M. A. Allodi, E. C. Martin, C. N. Hunter, G. S. Engel, Mapping the ultrafast flow of harvested solar energy in living photosynthetic cells. *Nat. Commun.* **8**, 988 (2017).

6. M. L. Cartron, J. D. Olsen, M. Sener, P. J. Jackson, A. A. Brindley, P. Qian, M. J. Dickman, G. J. Leggett, K. Schulten, C. N. Hunter, Integration of energy and electron transfer processes in the photosynthetic membrane of *Rhodobacter sphaeroides*. *Biochim. Biophys. Acta Bioenerg.* **1837**, 1769–1780 (2014).

7. A. Singharoy, C. Maffeo, K. H. Delgado-Magnero, D. J. K. Swainsbury, M. Sener, U. Kleinekathöfer, J. W. Vant, J. Nguyen, A. Hitchcock, B. Isralewitz, I. Teo, D. E. Chandler, J. E. Stone, J. C. Phillips, T. V. Pogorelov, M. I. Mallus, C. Chipot, Z. Luthey-Schulten, D. P. Tieleman, C. N. Hunter, E. Tajkhorshid, A. Aksimentiev, K. Schulten, Atoms to phenotypes: Molecular design principles of cellular energy metabolism. *Cell* **179**, 1098–1111.e23 (2019).

8. P. A. Bullough, P. Qian, C. N. Hunter, Reaction center-light-harvesting core complexes of purple bacteria, in *The Purple Phototrophic Bacteria*, C. N. Hunter, F. Daldal, M. C. Thurnauer, J. T. Beatty, Eds. (Springer, 2009), pp. 155–179.

9. L.-J. Yu, M. Suga, Z.-Y. Wang-Otomo, J.-R. Shen, Structure of photosynthetic LH1–RC supercomplex at 1.9 Å resolution. *Nature* **556**, 209–213 (2018).

10. P. Qian, C. A. Siebert, P. Wang, D. P. Canniffe, C. N. Hunter, Cryo-EM structure of the *Blastochloris viridis* LH1–RC complex at 2.9 Å. *Nature* **556**, 203–208 (2018).

11. A. T. Gardiner, T. C. Nguyen-Phan, R. J. Cogdell, A comparative look at structural variation among RC-LH1 'Core' complexes present in anoxygenic phototrophic bacteria. *Photosynth. Res.* **145**, 83–96 (2020).

12. K. Tani, R. Kanno, Y. Makino, M. Hall, M. Takenouchi, M. Imanishi, L.-J. Yu, J. Overmann, M. T. Madigan, Y. Kimura, A. Mizoguchi, B. M. Humbel, Z.-Y. Wang-Otomo, Cryo-EM structure of a Ca^{2+}-bound photosynthetic LH1-RC complex containing multiple αβ-polypeptides. *Nat. Commun.* **11**, 4955 (2020).

13. P. Qian, M. Z. Papiz, P. J. Jackson, A. A. Brindley, I. W. Ng, J. D. Olsen, M. J. Dickman, P. A. Bullough, C. N. Hunter, Three-dimensional structure of the *Rhodobacter sphaeroides* RC-LH1-PufX complex: Dimerization and quinone channels promoted by PufX. *Biochemistry* **52**, 7575–7585 (2013).

14. A. W. Roszak, T. D. Howard, J. Southall, A. T. Gardiner, C. J. Law, N. W. Isaacs, R. J. Cogdell, Crystal structure of the RC-LH1 core complex from *Rhodopseudomonas palustris*. *Science* **302**, 1969–1972 (2003).

15. Y. Xin, Y. Shi, T. Niu, Q. Wang, W. Niu, X. Huang, W. Ding, L. Yang, R. E. Blankenship, X. Xu, F. Sun, Cryo-EM structure of the RC-LH core complex from an early branching photosynthetic prokaryote. *Nat. Commun.* **9**, 1568 (2018).

16. P. J. Jackson, A. Hitchcock, D. J. K. Swainsbury, P. Qian, E. C. Martin, D. A. Farmer, M. J. Dickman, D. P. Canniffe, C. N. Hunter, Identification of protein W, the elusive sixth subunit of the *Rhodopseudomonas palustris* reaction center-light harvesting 1 core complex. *Biochim. Biophys. Acta Bioenerg.* **1859**, 119–128 (2018).

17. T. Mizoguchi, J. Harada, M. Isaji, S. Yoshida, H. Oh-oka, H. Tamiaki, Preferential binding of hydroxy-carotenoids to the peripheral antenna and methoxy-analogs to the core complexes in the purple photosynthetic bacterium *Rhodopseudomonas* sp. Rits. *Car. Sci.* **13**, 33–37 (2008).

18. F. Ma, L.-J. Yu, Z.-Y. Wang-Otomo, R. van Grondelle, The origin of the unusual Q_y red shift in LH1–RC complexes from purple bacteria *Thermochromatium tepidum* as revealed by Stark absorption spectroscopy. *Biochim. Biophys. Acta Bioenerg.* **1847**, 1479–1486 (2015).

19. P. McGlynn, W. H. J. Westerhuis, M. R. Jones, C. N. Hunter, Consequences for the organization of reaction center-light harvesting antenna 1 (LH1) core complexes of *Rhodobacter sphaeroides* arising from deletion of amino acid residues from the C terminus of the LH1 α polypeptide. *J. Biol. Chem.* **271**, 3285–3292 (1996).

20. S. Georgakopoulou, R. van Grondelle, G. van der Zwan, Explaining the visible and near-infrared circular dichroism spectra of light-harvesting 1 complexes from purple bacteria: A modeling study. *J. Phys. Chem. B* **110**, 3344–3353 (2006).

21. W. H. J. Westerhuis, J. N. Sturgis, E. C. Ratcliffe, C. N. Hunter, R. A. Niederman, Isolation, size estimates, and spectral heterogeneity of an oligomeric series of light-harvesting 1 complexes from *Rhodobacter sphaeroides*. *Biochemistry* **41**, 8698–8707 (2002).

22. S. Niwa, L.-J. Yu, K. Takeda, Y. Hirano, T. Kawakami, Z.-Y. Wang-Otomo, K. Miki, Structure of the LH1–RC complex from *Thermochromatium tepidum* at 3.0 Å. *Nature* **508**, 228–232 (2014).

23. K. R. Fixen, Y. Oda, C. S. Harwood, Redox regulation of a light-harvesting antenna complex in an anoxygenic phototroph. *MBio* **10**, e02838–19 (2019).

24. K. E. McAuley, P. K. Fyfe, J. P. Ridge, N. W. Isaacs, R. J. Cogdell, M. R. Jones, Structural details of an interaction between cardiolipin and an integral membrane protein. *Proc. Natl. Acad. Sci. U.S.A.* **96**, 14706–14711 (1999).

25. T. Mizoguchi, M. Isaji, J. Harada, H. Tamiaki, Identification of 3,4-didehydrorhodopin as major carotenoid in *Rhodopseudomonas* species. *Photochem. Photobiol. Sci.* **7**, 492–497 (2008).

26. M. H. B. Stowell, T. M. McPhillips, D. C. Rees, S. M. Soltis, E. Abresch, G. Feher, Light-induced structural changes in photosynthetic reaction center: Implications for mechanism of electron-proton transfer. *Science* **276**, 812–816 (1997).

27. C. R. D. Lancaster, H. Michel, The coupling of light-induced electron transfer and proton uptake as derived from crystal structures of reaction centres from *Rhodopseudomonas viridis* modified at the binding site of the secondary quinone, Q_B. *Structure* **5**, 1339–1359 (1997).

28. T. Nogi, I. Fathir, M. Kobayashi, T. Nozawa, K. Miki, Crystal structures of photosynthetic reaction center and high-potential iron-sulfur protein from *Thermochromatium tepidum*: Thermostability and electron transfer. *Proc. Natl. Acad. Sci. U.S.A.* **97**, 13561–13566 (2000).

29. G. Katona, U. Andréasson, E. M. Landau, L.-E. Andréasson, R. Neutze, Lipidic cubic phase crystal structure of the photosynthetic reaction centre from *Rhodobacter sphaeroides* at 2.35Å resolution. *J. Mol. Biol.* **331**, 681–692 (2003).

30. H. L. Axelrod, E. C. Abresch, M. Y. Okamura, A. P. Yeh, D. C. Rees, G. Feher, X-ray structure determination of the cytochrome c_2: Reaction center electron transfer complex from *Rhodobacter sphaeroides*. *J. Mol. Biol.* **319**, 501–515 (2002).

31. F. Francia, M. Dezi, A. Rebecchi, A. Mallardi, G. Palazzo, B. A. Melandri, G. Venturoli, Light-harvesting complex 1 stabilizes $P^+Q_B^-$ charge separation in reaction centers of *Rhodobacter sphaeroides*. *Biochemistry* **43**, 14199–14210 (2004).

32. F. Comayras, C. Jungas, J. Lavergne, Functional consequences of the organization of the photosynthetic apparatus in *Rhodobacter sphaeroides.* I. Quinone domains and excitation transfer in chromatophores and reaction center·antenna complexes. *J. Biol. Chem.* **280**, 11203–11213 (2005).

33. R. J. Shopes, C. A. Wraight, The acceptor quinone complex of *Rhodopseudomonas viridis* reaction centers. *Biochim. Biophys. Acta Bioenerg.* **806**, 348–356 (1985).

34. L. M. P. Beekman, R. W. Visschers, R. Monshouwer, M. Heer-Dawson, T. A. Mattioli, P. McGlynn, C. N. Hunter, B. Robert, I. H. M. van Stokkum, R. van Grondelle, M. R. Jones, Time-resolved and steady-state spectroscopic analysis of membrane-bound reaction centers from *Rhodobacter*

sphaeroides. Comparisons with detergent-solubilized complexes. *Biochemistry* **34**, 14712–14721 (1995).

35. D. J. K. Swainsbury, S. Scheidelaar, R. van Grondelle, J. A. Killian, M. R. Jones, Bacterial reaction centers purified with styrene maleic acid copolymer retain native membrane functional properties and display enhanced stability. *Angew. Chem. Int. Ed.* **53**, 11803–11807 (2014).

36. D. Kleinfeld, M. Y. Okamura, G. Feher, Electron-transfer kinetics in photosynthetic reaction centers cooled to cryogenic temperatures in the charge-separated state: Evidence for light-induced structural changes. *Biochemistry* **23**, 5780–5786 (1984).

37. J. Koepke, E.-M. Krammer, A. R. Klingen, P. Sebban, G. M. Ullmann, G. Fritzsch, pH modulates the quinone position in the photosynthetic reaction center from *Rhodobacter sphaeroides* in the neutral and charge separated states. *J. Mol. Biol.* **371**, 396–409 (2007).

38. T. Cardona, A. Sedoud, N. Cox, A. W. Rutherford, Charge separation in photosystem II: A comparative and evolutionary overview. *Biochim. Biophys. Acta Bioenerg.* **1817**, 26–43 (2012).

39. F. Reifarth, G. Renger, Indirect evidence for structural changes coupled with $Q_B^{-\bullet}$ formation in photosystem II. *FEBS Lett.* **428**, 123–126 (1998).

40. A. Garbers, F. Reifarth, J. Kurreck, G. Renger, F. Parak, Correlation between protein flexibility and electron transfer from $Q_A^{-\bullet}$ to Q_B in PSII membrane fragments from spinach. *Biochemistry* **37**, 11399–11404 (1998).

41. C. N. Hunter, G. Turner, Transfer of genes coding for apoproteins of reaction centre and light-harvesting LH1 complexes to *Rhodobacter sphaeroides*. *J. Gen. Microbiol.* **134**, 1471–1480 (1988).

42. J. Zivanov, T. Nakane, B. O. Forsberg, D. Kimanius, W. J. H. Hagen, E. Lindahl, S. H. W. Scheres, New tools for automated high-resolution cryo-EM structure determination in RELION-3. *eLife* **7**, e42166 (2018).

43. S. Q. Zheng, E. Palovcak, J.-P. Armache, K. A. Verba, Y. Cheng, D. A. Agard, MotionCor2: Anisotropic correction of beam-induced motion for improved cryo-electron microscopy. *Nat. Methods* **14**, 331–332 (2017).

44. A. Rohou, N. Grigorieff, CTFFIND4: Fast and accurate defocus estimation from electron micrographs. *J. Struct. Biol.* **192**, 216–221 (2015).

45. A. Waterhouse, M. Bertoni, S. Bienert, G. Studer, G. Tauriello, R. Gumienny, F. T. Heer, T. A. P. de Beer, C. Rempfer, L. Bordoli, R. Lepore, T. Schwede, SWISS-MODEL: Homology modelling of protein structures and complexes. *Nucleic Acids Res.* **46**, W296–W303 (2018).

46. V. M. Friebe, D. J. K. Swainsbury, P. K. Fyfe, W. van der Heijden, M. R. Jones, R. N. Frese, On the mechanism of ubiquinone mediated photocurrent generation by a reaction center based photocathode. *Biochim. Biophys. Acta Bioenerg.* **1857**, 1925–1934 (2016).

47. E. F. Pettersen, T. D. Goddard, C. C. Huang, G. S. Couch, D. M. Greenblatt, E. C. Meng, T. E. Ferrin, UCSF Chimera—A visualization system for exploratory research and analysis. *J. Comput. Chem.* **25**, 1605–1612 (2004).

48. P. Emsley, B. Lohkamp, W. G. Scott, K. Cowtan, Features and development of *Coot*. *Acta Crystallogr. Sect. D* **66**, 486–501 (2010).

49. P. D. Adams, P. V. Afonine, G. Bunkóczi, V. B. Chen, I. W. Davis, N. Echols, J. J. Headd, L.-W. Hung, G. J. Kapral, R. W. Grosse-Kunstleve, A. J. McCoy, N. W. Moriarty, R. Oeffner, R. J. Read, D. C. Richardson, J. S. Richardson, T. C. Terwilliger, P. H. Zwart, *PHENIX*: A comprehensive Python-based system for macromolecular structure solution. *Acta Crystallogr. Sect. D* **66**, 213–221 (2010).

50. A. J. Jakobi, M. Wilmanns, C. Sachse, Model-based local density sharpening of cryo-EM maps. *eLife* **6**, e27131 (2017).

51. H. P. Permentier, K. A. Schmidt, M. Kobayashi, M. Akiyama, C. Hager-Braun, S. Neerken, M. Miller, J. Amesz, Composition and optical properties of reaction centre core complexes from the

green sulfur bacteria *Prosthecochloris aestuarii* and *Chlorobium tepidum*. *Photosynth. Res.* **64**, 27–39 (2000).

52. D. J. K. Swainsbury, M. S. Proctor, A. Hitchcock, M. L. Cartron, P. Qian, E. C. Martin, P. J. Jackson, J. Madsen, S. P. Armes, C. N. Hunter, Probing the local lipid environment of the *Rhodobacter sphaeroides* cytochrome bc_1 and *Synechocystis* sp. PCC 6803 cytochrome b_6f complexes with styrene maleic acid. *Biochim. Biophys. Acta Bioenerg.* **1859**, 215–225 (2018).

53. D. J. K. Swainsbury, E. C. Martin, C. Vasilev, P. S. Parkes-Loach, P. A. Loach, C. N. Hunter, Engineering of a calcium-ion binding site into the RC-LH1-PufX complex of *Rhodobacter sphaeroides* to enable ion-dependent spectral red-shifting. *Biochim. Biophys. Acta Bioenerg.* **1858**, 927–938 (2017).

54. Y. Lin, L. Huo, Z. Liu, J. Li, Y. Liu, Q. He, X. Wang, S. Liang, Sodium laurate, a novel protease- and mass spectrometry-compatible detergent for mass spectrometry-based membrane proteomics. *PLOS ONE* **8**, e59779 (2013).

55. C. MacGregor-Chatwin, P. J. Jackson, M. Sener, J. W. Chidgey, A. Hitchcock, P. Qian, G. E. Mayneord, M. P. Johnson, Z. Luthey-Schulten, M. J. Dickman, D. J. Scanlan, C. N. Hunter, Membrane organization of photosystem I complexes in the most abundant phototroph on Earth. *Nat. Plants* **5**, 879–889 (2019).

56. J. Cox, M. Mann, MaxQuant enables high peptide identification rates, individualized p.p.b.-range mass accuracies and proteome-wide protein quantification. *Nat. Biotechnol.* **26**, 1367–1372 (2008).

57. M. D. Abràmoff, P. J. Magalhães, S. J. Ram, Image processing with ImageJ. *Biophotonics Int.* **11**, 36–41 (2004).

58. Y. Umena, K. Kawakami, J.-R. Shen, N. Kamiya, Crystal structure of oxygen-evolving photosystem II at a resolution of 1.9 Å. *Nature* **473**, 55–60 (2011).

59. V. Šlouf, P. Chábera, J. D. Olsen, E. C. Martin, P. Qian, C. N. Hunter, T. Polívka, Photoprotection in a purple phototrophic bacterium mediated by oxygen-dependent alteration of carotenoid excited-state properties. *Proc. Natl. Acad. Sci. U.S.A.* . **109**, 8570–8575 (2012).

60. K. M. Faries, N. P. Dylla, D. K. Hanson, D. Holten, P. D. Laible, C. Kirmaier, Manipulating the energetics and rates of electron transfer in *Rhodobacter capsulatus* reaction centers with asymmetric pigment content. *J. Phys. Chem. B* **121**, 6989–7004 (2017).
